# Clinical Utility of Urinary Cystatin C in Early Screening and Staging of Diabetic Kidney Disease in Type 2 Diabetes

**DOI:** 10.1155/ije/8881466

**Published:** 2026-04-22

**Authors:** Shanshan Ji, Fanghao Shang, Bingbing Chen, Wentao Tang, Hua Fang, Jinbao Huang, Yizhong Hu

**Affiliations:** ^1^ Department of Laboratory Medicine, The People’s Hospital of Chizhou, Chizhou, China

**Keywords:** diabetic kidney disease, early renal injury biomarkers, noninvasive diagnosis, Type 2 diabetes mellitus, urinary cystatin C

## Abstract

**Objectives:**

Diabetic kidney disease (DKD) is a major complication of Type 2 diabetes mellitus (T2DM). Early detection is critical to prevent renal function decline and improve outcomes, but conventional biomarkers (e.g., urinary albumin‐to‐creatinine ratio [UACR] and estimated glomerular filtration rate [eGFR]) have limited sensitivity for early‐stage kidney damage. Urinary cystatin C (UcyC) is a protein marker of impaired tubular reabsorption and a promising biomarker for early tubulointerstitial injury in DKD. This study assessed the clinical utility of UcyC for early detection and staging of DKD and compared its diagnostic performance with that of serum cystatin C (CysC).

**Methods:**

This prospective study included 102 patients with T2DM who were enrolled at The People’s Hospital of Chizhou, China, between May 2022 and October 2025. The participants were classified as having T2DM without kidney disease (T2DM without KD) or DKD based on UACR and eGFR. The DKD group was divided into early DKD (EDKD) and clinical DKD (CDKD). Demographic and laboratory data were obtained from the hospital’s medical records. Multivariate logistic regression analyses independent risk factors of DKD. Correlation analysis and receiver operating characteristic (ROC) curve analyses assess diagnostic accuracy and disease staging effectiveness of DKD.

**Results:**

Compared with T2DM individuals free of renal complications, DKD patients showed significantly elevated UcyC concentrations (*p* < 0.001), paralleling advancing kidney impairment severity. Logistic regression quantified > 350‐fold higher DKD risk per 10‐fold logUcyC increase. UcyC demonstrated strong positive correlation with UACR and inverse correlation with eGFR, reinforcing its utility for tracking disease progression. Notably, ROC analysis confirmed superior staging accuracy versus CysC (AUC 0.830 vs. 0.691) and enhanced sensitivity plus negative predictive value for early DKD detection.

**Conclusion:**

UcyC, a noninvasive and highly sensitive biomarker for renal injury, could be valuable for early screening, assessing risk levels, and potentially guiding treatment personalization in DKD. This may streamline diagnosis and help tailor management plans for patients with DKD.

## 1. Introduction

Diabetic kidney disease (DKD), a common microvascular pathology, is recognized as one of the most prevalent and clinically impactful chronic complications of Type 2 diabetes mellitus (T2DM) [1, 2]. The International Diabetes Federation reports that DKD in roughly one‐third of its estimated 537 million adults with diabetes globally [3]. With the escalating diabetes pandemic, DKD now drives increasing cases of end‐stage renal disease (ESRD) [4]. Upon reaching advanced DKD stages, established lesions like glomerulosclerosis and tubulointerstitial fibrosis preclude structural recovery, leaving few clinically validated treatment options [1, 5, 6]. Hence, capturing subclinical DKD—defined by initiating mesangial matrix deposition, podocyte stress, and subclinical tubulointerstitial remodeling—represents an actionable window for nephroprotection. Precise biomarker‐guided intervention at this stage curtails the self‐perpetuating cycle of renal deterioration, functionally delaying kidney failure progression and fundamentally improving survival trajectories.

Urinary albumin‐to‐creatinine ratio (UACR) and estimated glomerular filtration rate (eGFR) are the primary tools for the clinical screening of DKD [7]. Notably, UACR’s capacity to identify incipient DKD proves critically inadequate: histologically confirmed podocyte effacement and mesangiolysis frequently evolve during periods of deceptively normative albumin excretion, systematically generating false assurance [8]. Similarly, although eGFR is a standard indicator of glomerular filtration, its reliance on serum creatinine (CREA) levels renders it vulnerable to nonrenal influences, including muscle mass, dietary intake, and changes in body weight [9, 10]. Furthermore, a measurable decline in eGFR typically occurs only after significant renal damage, limiting its effectiveness in detecting ESRD.

These limitations underscore the urgent need for biomarkers that can reliably detect kidney damage at the early stages of DKD. In this regard, urinary cystatin C (UcyC) is a low‐molecular‐weight cysteine protease inhibitor secreted by all nucleated cells. It is freely filtered by the glomerulus and subsequently reabsorbed and catabolized by the proximal tubules. When tubular reabsorption is impaired, UcyC concentrations increase, supporting its potential as a promising protein biomarker of kidney function. UcyC levels are closely associated with renal function and may reflect early pathological changes in the kidney, as reported in recent studies examining the pathophysiology and biomarker profiles of DKD [10]. Evidence suggests that elevated UcyC levels are associated with kidney injury, even before detectable changes in eGFR and UACR are observed, highlighting its utility as an early diagnostic marker for DKD, particularly in asymptomatic individuals in whom renal dysfunction may not be captured by conventional markers.

Although UcyC has demonstrated clinical utility for diagnosing chronic kidney disease (CKD) and acute kidney injury (AKI) [11–13], its applicability in the early detection and staging of DKD remains underexplored. Therefore, this study aims to evaluate the diagnostic performance of UcyC, alongside that of other biomarkers, across the clinical stages of Type 2 DKD. By examining their potential as early screening indicators, this study seeks to inform early risk stratification efforts and improve prognosis in patients with DKD.

## 2. Materials and Methods

### 2.1. Study Population

This prospective study was conducted at The People’s Hospital of Chizhou, Anhui Province, China, with recruitment spanning from May 2022 to October 2025. A total of 370 individuals meeting established diagnostic criteria for diabetes were initially identified [14]. To ensure cohort homogeneity and analytical rigor, we systematically applied predefined inclusion and exclusion criteria [15]. Participants were excluded if they met any of the following conditions: (1) Type 1 diabetes mellitus or other specific forms of diabetes; (2) acute diabetic complications or severe chronic diabetic complications; (3) heart failure, unstable angina, severe hypertension, arrhythmias, or myocardial or cerebral infarction in the preceding 12 months; (4) pregnancy or lactation; (5) diagnosed autoimmune disease or malignancy; (6) a history of recurrent urinary tract infections; (7) clinically significant thyroid dysfunction, defined as thyroid‐stimulating hormone (TSH) levels < 0.35 μIU/mL or > 4.94 μIU/mL or abnormal free thyroxine (FT4) levels; (8) insufficient baseline data, defined as more than 20% missing core parameters, including body mass index (BMI), glycated hemoglobin A_1c_ (HbA_1c_), renal function indicators, or medication records; and (9) other medical conditions known to affect renal function.

**FIGURE 1:**
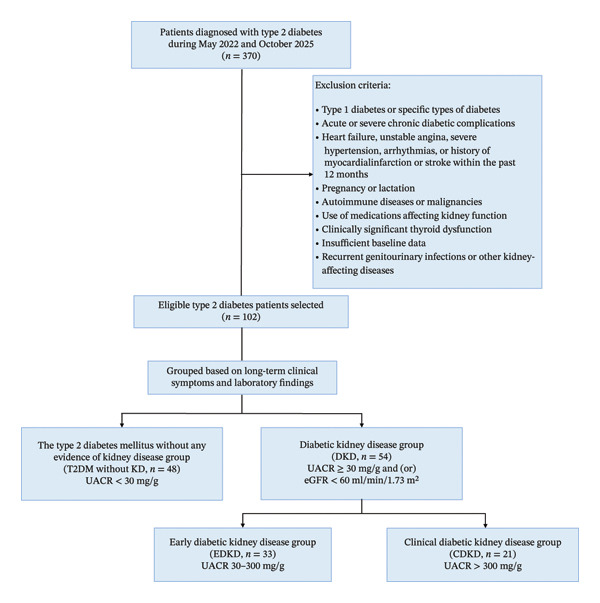
Flowchart of participant inclusion and subgroup classification for DKD disease in the study.

After applying these criteria, 102 individuals diagnosed with T2DM were enrolled in the study. Based on long‐term clinical follow‐up and laboratory data, the participants were classified into two subgroups in line with the latest clinical practice guidelines for diabetes management [15]: a T2DM without kidney disease (T2DM without KD) group (*n* = 48) and a DKD group (*n* = 54). T2DM without KD was defined as a sustained UACR < 30 mg/g and an eGFR ≥ 60 mL/min/1.73 m^2^ documented across at least three follow‐up visits over a period exceeding 3 months, excluding other causes of proteinuria or CKD via urinalysis, urinary sediment examination, and clinical history review. DKD was diagnosed when at least three follow‐up assessments revealed a UACR ≥ 30 mg/g or an eGFR < 60 mL/min/1.73 m^2^, with abnormalities persisting for more than 3 months and when other causes of proteinuria or CKD could be excluded. The DKD group was further classified according to disease stage into early DKD (EDKD; *n* = 33, UACR 30–300 mg/g) and clinical DKD (CDKD; *n* = 21, UACR > 300 mg/g). The processes for participant selection and grouping are illustrated in Figure 1. The study protocol was approved by the Ethics Committee of The People’s Hospital of Chizhou (Approval No. 2021‐KY‐06).

### 2.2. Data Collection

Patient demographics, clinical characteristics, laboratory results, and diagnostic data were systematically extracted from the hospital’s electronic medical record system by trained medical personnel. Laboratory assessments included fasting C‐peptide (FCP), 2‐h postprandial C‐peptide (2hPP‐CP), fasting blood glucose (FBG), 2‐h postprandial blood glucose (2hPBG), HbA_1c_, total protein (TP), albumin (ALB), total bilirubin (TBIL), direct bilirubin (DBIL), aspartate aminotransferase (AST), alanine aminotransferase (ALT), gamma‐glutamyl transferase (GGT), alkaline phosphatase (ALP), urea, CREA, uric acid (UA), eGFR, CysC, urinary creatinine (UCREA), urinary microalbumin (UMALB), UACR, UcyC, triglycerides (TG), total cholesterol (TC), high‐density lipoprotein cholesterol (HDL‐C), low‐density lipoprotein cholesterol (LDL‐C), apolipoprotein A1 (ApoA1), apolipoprotein B (ApoB), apolipoprotein E (ApoE), nonesterified fatty acids (NEFA), lipoprotein (a) (Lp[a]), free triiodothyronine (FT3), free thyroxine (FT4), thyroid‐stimulating hormone (TSH), white blood cell count (WBC), neutrophil count (Neut#), lymphocyte count (Lym#), red blood cell count (RBC), hemoglobin (Hb), and platelet count (PLT). UcyC levels were quantified using latex immunoturbidimetry, whereas UMALB and UCREA were measured using standardized quantitative methods. UACR was calculated as UMALB (mg/L) divided by UCREA (mmol/L) multiplied by 113 to yield units of mg/g, reflecting glomerular filtration barrier function; this conversion standardizes the molecular weight of creatinine (113 g/mol). eGFR was calculated via the modified CKD Epidemiology Collaboration (CKD‐EPI) equation incorporating CREA levels [16], a formulation validated for estimating global glomerular filtration capacity in diabetic cohorts. To guarantee data credibility for downstream statistical analyses, all values underwent dual independent verification by trained researchers.

### 2.3. Statistical Analysis

Continuous data analysis employed distribution‐adaptive protocols. For comparisons between two groups, normally distributed variables were assessed using Student’s *t*‐test and reported as mean ± standard deviation (SD). In contrast, non‐normally distributed variables were analyzed using the Mann–Whitney *U* test and reported as median interquartile range (IQR). For comparisons across three or more groups, normally distributed variables were analyzed using one‐way analysis of variance followed by Bonferroni post hoc tests, whereas non‐normally distributed variables were analyzed using the Kruskal–Wallis *H* test with Dunn’s post hoc test and Bonferroni correction. Categorical variables were examined using the Chi‐square test and expressed as counts and percentages [*n* (%)]. Associations between variables were assessed using Spearman’s rank correlation coefficient.

Potential risk factors for DKD were screened using univariate logistic regression, followed by multivariate logistic regression to identify independent predictors. Receiver operating characteristic (ROC) curve analysis was performed to evaluate diagnostic performance by calculating the area under the curve (AUC) calculated. The optimal cutoff value was determined using the Youden index, and sensitivity, specificity, diagnostic accuracy, positive predictive value (PPV), and negative predictive value (NPV) were subsequently computed. All statistical analyses and data visualizations were conducted using R software (version 4.3.2). A two‐tailed *p* value < 0.05 was considered statistically significant.

## 3. Results

### 3.1. Demographic and Clinical Characteristics of the Patients

This prospective cohort included 102 Type 2 diabetes patients, categorized by renal involvement into two groups: 48 subjects with Type 2 diabetes without kidney disease and 54 patients with a definitive diagnosis of DKD. Table 1 outlines the baseline characteristics of the cohort, which was predominantly male (53.9%, *n* = 55) and had an overall mean age of 60.50 ± 11.51 years.

**TABLE 1:** Baseline characteristics of 102 patients with T2DM.

Characteristics	Overall (*n* = 102)	T2DM without KD (*n* = 48)	DKD (*n* = 54)	*p* value
*Demographic data*				
Sex (male), *n* (%)	55 (53.9)	27 (56.3)	28 (51.9)	0.656
Age (year), mean ± SD	60.50 ± 11.51	59.17 ± 11.53	61.69 ± 11.50	0.272
BMI (kg/m^2^), mean ± SD	24.64 ± 3.71	24.30 ± 3.53	24.93 ± 3.86	0.394
SBP (mm/Hg), median (IQR)	137 (122, 150)	135 (120, 145)	142 (128, 151)	0.054
DBP (mm/Hg), median (IQR)	82 (75, 88)	80 (71, 85)	83 (78, 89)	**0.049**
Smoking (yes), *n* (%)	20 (22.5)	7 (14.6)	13 (24.1)	0.228
Alcohol (yes), *n* (%)	25 (24.5)	10 (20.8)	15 (27.8)	0.416
Diabetes duration (year), median (IQR)	7.0 (2.0, 11.8)	5.0 (0.5, 14.3)	8.0 (3.8, 10.3)	0.083
Family history of diabetes mellitus (yes), *n* (%)	22 (21.6)	10 (20.8)	12 (22.2)	1.000

*Past medical history (yes), n (%)*				
Hypertension	62 (60.8)	22 (45.8)	40 (74.1)	**0.004**
Cerebral infarction	10 (9.8)	4 (8.3)	6 (11.1)	0.746
Hyperlipidemia	42 (31.4)	16 (33.3)	16 (29.6)	0.687
History of HBV infection	7 (6.9)	5 (10.4)	2 (3.7)	0.249
History of cataract	13 (12.7)	6 (12.5)	7 (13.0)	0.994

*Macrovascular/microvascular diabetic complications (yes), n (%)*				
Peripheral arterial disease	67 (65.7)	28 (58.3)	39 (72.2)	0.140
Diabetic peripheral neuropathy	43 (42.2)	18 (37.5)	25 (46.3)	0.369
Diabetic retinopathy	7 (6.9)	0 (0.0)	7 (13.0)	**0.014**

*Clinical symptoms (yes), n (%)*				
Xerostomia	76 (74.5)	37 (77.1)	39 (72.2)	0.574
Polydipsia	72 (70.6)	36 (75.0)	36 (66.7)	0.357
Polyuria	75 (73.5)	32 (66.7)	43 (79.5)	0.139
Unintended weight loss	37 (36.3)	19 (39.6)	18 (33.3)	0.512
Paresthesia	38 (37.3)	15 (31.3)	23 (42.6)	0.237
Visual impairment	16 (15.7)	8 (16.7)	8 (14.8)	0.797
Blurred vision	46 (45.1)	19 (39.6)	27 (50.0)	0.291
Lower extremity edema	6 (5.9)	2 (4.2)	4 (7.4)	0.681
Skin pruritus	3 (2.9)	2 (4.2)	1 (1.9)	0.600
Chest discomfort	5 (4.9)	2 (4.2)	3 (5.6)	1.000
Fatigue	26 (25.5)	12 (25.0)	14 (25.9)	0.915

*Current medication status (yes), n (%)*				
ACEI	5 (4.9)	4 (8.3)	1 (1.9)	0.185
ARB	16 (15.7)	7 (14.6)	9 (16.7)	0.773
SGLT‐2i	8 (7.8)	1 (2.1)	7 (13.0)	0.063
GLP‐1 RAs	2 (2.0)	0 (0.0)	2 (3.7)	0.497
Finerenone	0 (0.0)	0 (0.0)	0 (0.0)	—
TZDs	4 (3.9)	1 (2.1)	3 (5.6)	0.620
Insulin secretagogues	22 (21.6)	8 (16.7)	14 (25.9)	0.256
Insulin	31 (30.4)	13 (27.1)	18 (33.3)	0.493
Metformin	40 (39.2)	20 (41.7)	20 (37.0)	0.693
α‐Glucosidase inhibitors	9 (8.8)	5 (10.4)	4 (7.4)	0.731
Statins (yes), *n* (%)	12 (11.8)	7 (14.6)	5 (9.3)	0.405

*Note:* Data are presented as *n* (%), mean ± SD, or median (IQR). Normally distributed variables were compared using Student’s *t*‐test; skewed continuous variables with the Mann–Whitney *U* test; and categorical variables with the Chi‐square test or Fisher’s exact test, as appropriate. Statistically significant differences (*p* < 0.05) are highlighted in bold.

Abbreviations: ACEI, angiotensin‐converting enzyme inhibitors; ARB, angiotensin II receptor blockers; BMI, body mass index; DBP, diastolic blood pressure; GLP‐1 RAs, glucagon‐like peptide‐1 receptor agonists; HBV: hepatitis B virus; IQR, interquartile range; SBP, systolic blood pressure; SGLT‐2i, sodium‐glucose cotransporter 2 inhibitors; TZDs, thiazolidinediones.

When compared to the T2DM without KD group, the DKD cohort had a substantially elevated prevalence of hypertension (74.1% vs. 45.8%, Δ = 28.3%; *p* = 0.004). Diabetic retinopathy demonstrated complete penetrance exclusivity to DKD cases (13.0% vs. 0.0%, *p* = 0.014), and diastolic blood pressure (DBP) was elevated among DKD participants (83 vs. 80 mmHg, *p* = 0.049). In terms of baseline covariates, the two cohorts were well matched with respect to gender ratio, age, smoking and drinking histories, medication prescriptions, and the overall burden of other comorbidities, with no statistically significant disparities identified across these variables.

We analyzed the liver function, renal function, and various metabolic indices in two patient groups. Our findings indicated that, compared to the T2DM without kidney disease (KD) group, the renal function markers in the DKD group were significantly elevated. This was primarily reflected in the increased CREA level (82.90 vs. 68.25 μmol/L, *p* = 0.001) and the rise in CysC concentration (1.14 vs. 0.92 mg/L, *p* = 0.001). Additionally, UcyC levels were significantly higher (0.24 vs. 0.21 mg/L, *p* < 0.001). Among the liver function indices, ALP was significantly increased (91 vs. 77 U/L, *p* = 0.003). Furthermore, there was a trend toward an increase in the FBG level (10.50 vs. 8.78 mmol/L, *p* = 0.016). Although differences in urea levels were observed between the groups, it is important to note that urea levels can be easily influenced by various nonrenal factors such as protein intake, infections, and tumors. Therefore, we cannot definitively determine its relationship with DKD (Table 2).

**TABLE 2:** Clinical parameters of 102 eligible Type 2 diabetes patients included.

Parameters	Overall (*n* = 102)	T2DM without KD (*n* = 48)	DKD (*n* = 54)	*p* value
*Glucose metabolic indicators*				
FCP (ng/mL), median (IQR)	1.69 (1.07, 2.39)	1.46 (0.93, 2.05)	1.85 (1.47, 2.83)	0.052
2hPP‐CP (ng/mL), median (IQR)	3.47 (2.14, 5.35)	3.38 (2.26, 5.37)	3.64 (2.11, 6.14)	0.972
FBG (mmol/L), median (IQR)	9.54 (7.21, 12.44)	8.78 (6.61, 11.37)	10.50 (7.98, 14.46)	**0.016**
2hPBG (mmol/L), mean ± SD	16.84 ± 5.97	15.79 ± 5.13	17.87 ± 6.59	0.086
HbA_1c_ (%), median (IQR)	9.5 (7.5, 11.0)	9.4 (7.3, 10.5)	9.5 (8.1, 11.6)	0.221

*Hepatic function indicators*				
TP (g/L), mean ± SD	68.85 ± 7.33	68.04 ± 6.90	69.57 ± 7.68	0.296
ALB (g/L), mean ± SD	40.35 ± 4.83	41.21 ± 3.92	39.58 ± 5.44	0.083
TBIL (μmol/L), median (IQR)	11.95 (9.58, 15.03)	12.65 (11.05, 16.70)	10.65 (9.34, 13.20)	**0.006**
DBIL (μmol/L), median (IQR)	3.00 (2.50, 4.00)	3.35 (2.60, 4.20)	2.80 (2.43, 3.75)	0.071
AST (U/L), median (IQR)	19.00 (15.00, 25.75)	19.00 (17.00, 30.00)	19.00 (14.50, 23.00)	0.078
ALT (U/L), median (IQR)	19.00 (13.75, 30.50)	19.50 (16.00, 34.25)	18.50 (10.25, 28.50)	**0.049**
GGT (U/L), median (IQR)	23.00 (16.75, 47.00)	21.00 (14.75, 29.25)	28.50 (18.25, 58.75)	0.179
ALP (U/L), median (IQR)	85.00 (68.00, 102.00)	77.00 (58.00, 98.00)	91.00 (79.25, 105.00)	**0.003**

*Kidney function indicators*				
UREA (mmol/L), median (IQR)	5.81 (4.89, 7.32)	5.28 (4.71, 6.54)	6.34 (5.07, 7.88)	**0.005**
CREA (umol/L), median (IQR)	72.25 (59.00, 91.85)	68.25 (55.70, 79.70)	82.90 (62.10, 109.60)	**0.001**
UA (μmol/L), mean ± SD	325.29 ± 97.51	310.23 ± 84.15	338.69 ± 107.02	0.137
eGFR (ml/min/1.73 m^2^), mean ± SD	84.11 ± 23.46	94.51 ± 14.11	74.87 ± 26.23	**< 0.001**
CysC (mg/L), median (IQR)	1.01 (0.83, 1.27)	0.92 (0.83, 1.08)	1.14 (0.90, 1.56)	**0.001**
UCREA (mmol/L), median (IQR)	7.85 (5.40, 11.93)	7.60 (3.44, 12.36)	8.05 (5.60, 11.28)	0.393
UMALB (mg/L), median (IQR)	38.85 (11.50, 197.60)	11.05 (4.98, 16.45)	179.15 (65.13, 542.15)	**< 0.001**
UACR (mg/g), median (IQR)	34.65 (9.83, 193.73)	9.55 (5.50, 14.05)	160.10 (61.00, 519.02)	**< 0.001**
UcyC (mg/L), median (IQR)	0.22 (0.10, 0.24)	0.21 (0.06, 0.22)	0.24 (0.22, 0.28)	**< 0.001**

*Lipid metabolic indicators*				
TG (mmol/L), median (IQR)	1.43 (1.00, 2.39)	1.31 (0.98, 2.21)	1.54 (1.08, 2.50)	0.254
TC (mmol/L), mean ± SD	4.93 ± 1.18	4.90 ± 1.25	4.98 ± 1.13	0.845
HDL‐C (mmol/L), median (IQR)	1.17 (0.95, 1.50)	1.22 (0.98, 1.54)	1.08 (0.94, 1.40)	0.207
LDL‐C (mmol/L), mean ± SD	2.51 ± 0.81	2.49 ± 0.79	2.52 ± 0.83	0.833
ApoA1 (g/L), median (IQR)	1.40 (1.21, 1.58)	1.46 (1.22, 1.58)	1.36 (1.19, 1.57)	0.226
ApoB (g/L), mean ± SD	0.92 ± 0.25	0.89 ± 0.23	0.96 ± 0.26	0.155
ApoE (mg/dL), median (IQR)	2.75 (2.20, 3.70)	2.97 (2.26, 3.70)	2.65 (2.10, 3.62)	0.625
NEFA (mmol/L), median (IQR)	0.49 (0.33, 0.72)	0.49 (0.31, 0.71)	0.48 (0.34, 0.72)	0.882
Lp(a) (mg/L), median (IQR)	100.00 (50.00, 210.00)	76.00 (41.00, 186.00)	112.50 (53.75, 219.75)	0.229

*Thyroid function indicators*				
FT3 (pg/mL), mean ± SD	2.48 ± 0.46	2.50 ± 0.49	2.46 ± 0.43	0.628
FT4 (ng/dL), median (IQR)	0.98 (0.86, 1.09)	0.98 (0.85, 1.07)	0.98 (0.91, 1.11)	0.401
TSH (μIU/mL), median (IQR)	2.07 (1.51, 2.68)	2.01 (1.41, 2.79)	2.07 (1.78, 2.64)	0.845

*Hematology indicators*				
WBC (× 10^9^/L), median (IQR)	5.65 (4.88, 6.97)	5.51 (4.71, 6.32)	6.11 (5.00, 7.32)	0.050
Neut# (× 10^9^/L), median (IQR)	3.39 (2.59, 4.43)	3.01 (2.41, 3.94)	3.63 (2.84, 4.79)	**0.021**
Lym# (× 10^9^/L), mean ± SD	1.78 ± 0.56	1.82 ± 0.49	1.75 ± 0.63	0.513
RBC (× 10^12^/L), mean ± SD	4.42 ± 0.63	4.49 ± 0.53	4.36 ± 0.71	0.274
Hb (g/L), median (IQR)	131 (120, 145)	134 (122, 145)	131 (117, 144)	0.149
PLT (× 10^9^/L), median (IQR)	183.0 (137.5, 219.5)	183.0 (153.5, 202.8)	183.0 (123.5, 220.5)	0.862

*Note:* Data are presented as mean ± SD or median (IQR). Continuous variables were assessed using Student’s *t*‐test or Mann–Whitney *U* test, as appropriate. Statistically significant differences (*p* < 0.05) are highlighted in bold. UACR (mg/g) = [urinary albumin (mg/L)/urinary creatinine (mmol/L)] × 113, where 113 is the molecular weight of creatinine.

Abbreviations: 2hPBG, 2‐h postprandial blood glucose; 2hPP‐CP, 2‐h postprandial C‐peptide; ALB, albumin; ALP, alkaline phosphatase; ALT, alanine aminotransferase; ApoA1, apolipoprotein A1; ApoB, apolipoprotein B; ApoE, apolipoprotein E; AST, aspartate aminotransferase; CREA, creatinine; CysC, serum cystatin C; DBIL, direct bilirubin; eGFR, estimate glomerular filtration rate; FBG, fasting blood glucose; FCP, fasting C‐peptide; FT3, free triiodothyronine; FT4, free thyroxine; GGT, gamma‐glutamyl transferase; Hb, hemoglobin; HbA_1c_, glycated hemoglobin A_1c_; HDL‐C, high‐density lipoprotein cholesterol; IQR, interquartile range; LDL‐C, low‐density lipoprotein cholesterol; Lp(a), lipoprotein(a); Lym#, lymphocyte count; NEFA, non‐esterified fatty acids; Neut#, neutrophil count; PLT, platelet count; RBC, red blood cell count; SD, standard deviation; TBIL, total bilirubin; TC, total cholesterol; TG, triglycerides; TP, total protein; TSH, thyroid‐stimulating hormone; UA, uric acid; UACR, urine microalbumin/creatinine ratio; UCREA, urine creatinine; UcyC, urine cystatin C; UMALB, urine microalbumin; WBC, white blood cell count.

These findings support the clinical association of UcyC with renal pathology in T2DM. Its pronounced elevation parallels conventional biomarkers and specifically reflects tubular dysfunction. Although urea exhibits limited diagnostic specificity, the substantial differential in UcyC and CysC concentrations highlights its potential utility for the early detection of tubular injury across staged DKD populations.

### 3.2. Differences in Biomarkers Across Stages of DKD

Stratified analysis of biomarker trajectories across DKD stages showed that UcyC was the only marker with a consistent diagnostic value across the full spectrum of DKD (Figure 2(a)). UcyC increased significantly in the EDKD subgroup compared with the T2DM without KD group (0.23 vs. 0.21 mg/L, *p* < 0.001). This elevation persisted and increased further in the CDKD subgroup relative to the T2DM without KD group (*p* < 0.001), and UcyC also differentiated EDKD from CDKD (*p* = 0.048) (Supporting Table 1).

FIGURE 2Distribution of UcyC (a), CysC (b), ALB (c), TBIL (d), Lp(a) (e), ALP (f), FCP (g), and FBG (h) across stages of DKD. Data are presented as box plots, half‐violin plots, and scatter density plots; boxes represent the interquartile range, half‐violin plots depict data distribution, and scatter points represent individual observations. Ratios were calculated using geometric means. Global group differences were evaluated using one‐way analysis of variance or the Kruskal–Wallis *H* test, as appropriate. For each biomarker, three pairwise comparisons were performed, with Bonferroni correction applied to the significance thresholds; all reported p values were Bonferroni‐corrected. Results not reaching significance after correction are indicated as NS. Abbreviations: ALB, albumin; ALP, alkaline phosphatase; CysC, serum cystatin C; FBG, fasting blood glucose; FCP, fasting C‐peptide; Lp(a), lipoprotein(a); TBIL, total bilirubin; UcyC, urinary cystatin C.

**Figure:**
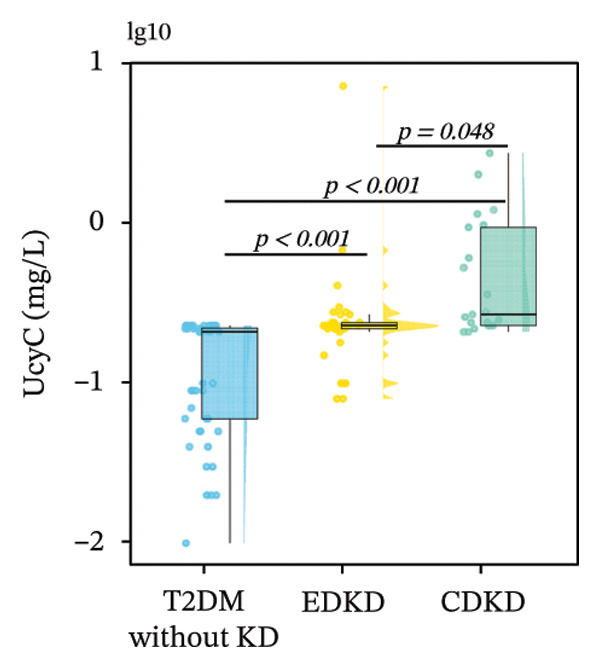
(a)

**Figure:**
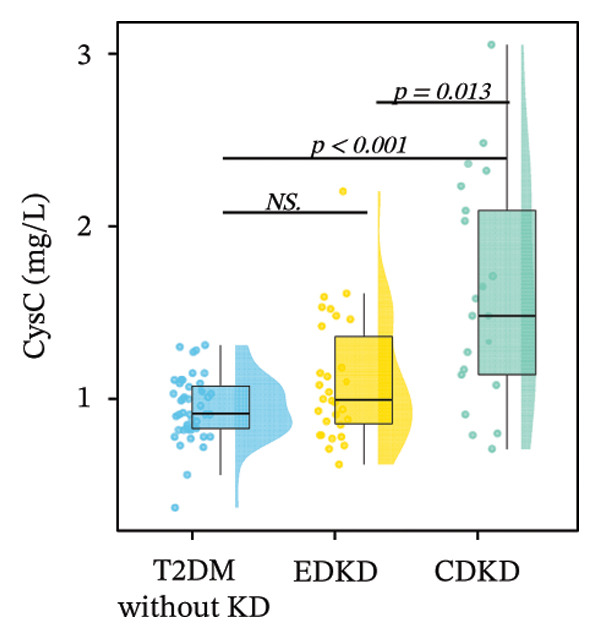
(b)

**Figure:**
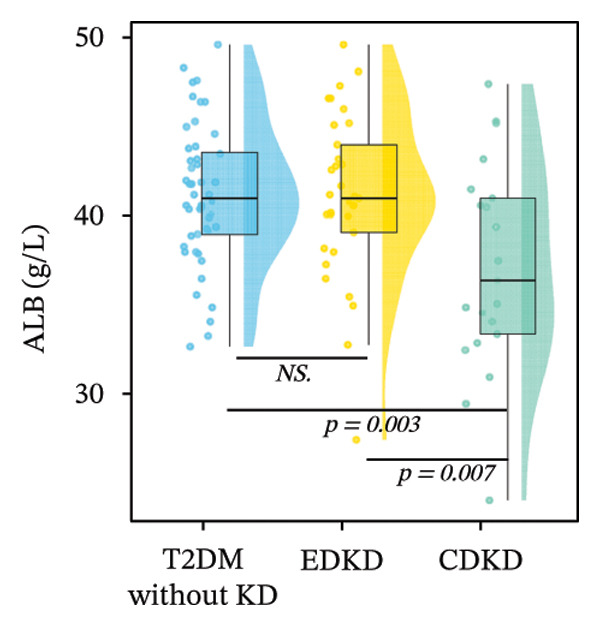
(c)

**Figure:**
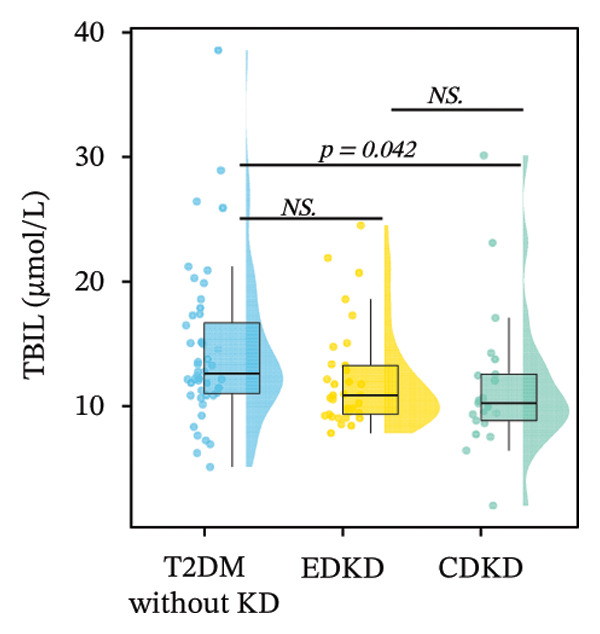
(d)

**Figure:**
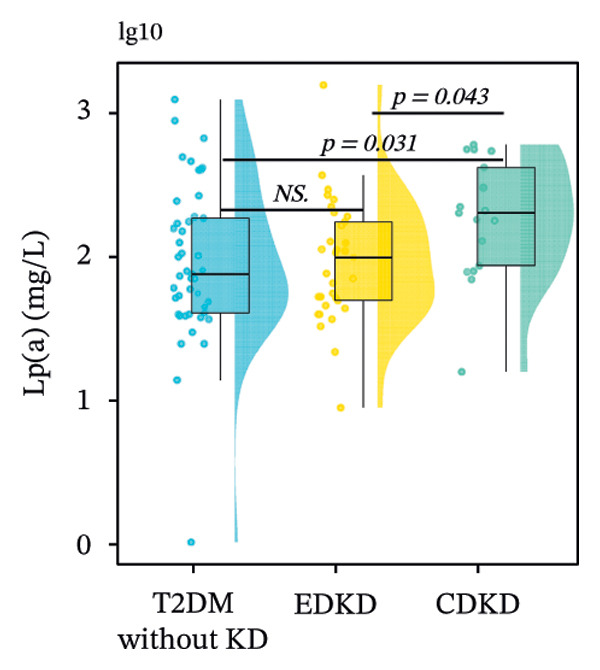
(e)

**Figure:**
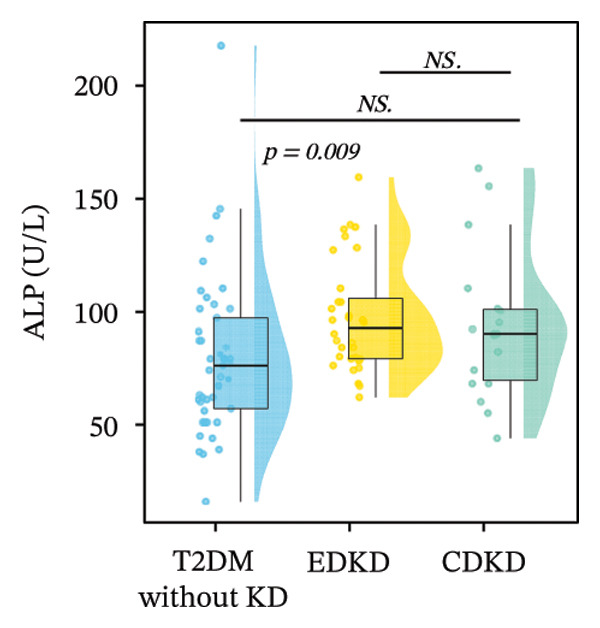
(f)

**Figure:**
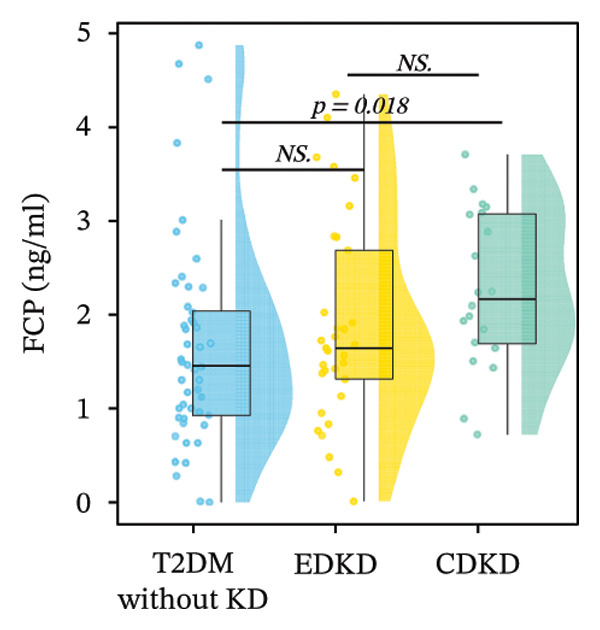
(g)

**Figure:**
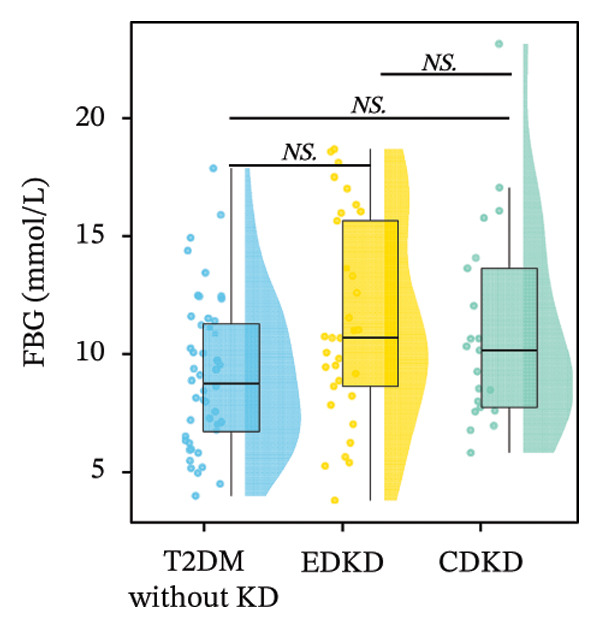
(h)

In contrast, the other biomarkers presented clear limitations in either or both early detection and progression monitoring. CysC did not distinguish EDKD from T2DM without KD (1.00 vs. 0.92 mg/L, *p* = 0.501), achieving statistical significance only at the CDKD stage (*p* < 0.001) (Figure 2(b)). ALB similarly lacked discriminatory power in early‐stage DKD (EDKD vs. T2DM without KD: 41.13 ± 4.62 vs. 41.21 ± 3.92 g/L, *p* = 0.997), showing a significant decline only in CDKD (*p* = 0.003) (Figure 2(c)). Meanwhile, Lp(a) failed to differentiate EDKD from T2DM without KD (*p* = 1.000) (Figure 2(e)). ALP significantly differentiated between EDKD and T2DM without KD (*p* = 0.009); however, it did not distinguish between EDKD and CDKD (*p* = 1.000) (Figure 2(f)). TBIL (*p* = 0.097) and FCP (*p* = 0.960) showed no capacity to detect EDKD (Figures 2(d) and 2(g)). FBG lost stage‐specific significance after statistical correction (Figure 2(h)).

Collectively, the findings indicate that UcyC fulfills three critical clinical functions in the management of DKD in T2DM: (1) sensitive early diagnosis (distinguishing EDKD from T2DM without KD, *p* < 0.001); (2) confirmation of advanced disease (identifying CDKD, *p* < 0.001); and (3) dynamic monitoring of progression (differentiating EDKD from CDKD, *p* = 0.048). This tripartite capability distinguishes UcyC from CysC, which lacks early diagnostic sensitivity, and from hepatometabolic markers, which remain nonsignificant across multiple disease stages.

### 3.3. Correlation Analysis With UACR and eGFR

We examined the relationships among UcyC, CysC, and key diagnostic markers for DKD, namely, UACR and eGFR. UcyC and CysC exhibited significant positive correlations with UACR (*r* = 0.545, *p* < 0.001; *r* = 0.442, *p* < 0.001, respectively) (Figures 3(c) and 3(d)). Conversely, UcyC exhibited a negative correlation with eGFR (*r* = −0.388, *p* < 0.001), and CysC showed a stronger inverse association with eGFR (*r* = −0.814, *p* < 0.001) (Figures 3(a) and 3(b)). These findings indicate that UcyC and CysC are closely associated with UACR and eGFR, supporting their relevance as biomarkers of kidney injury during DKD progression.

FIGURE 3Correlation analysis of UcyC and CysC with UACR and eGFR. CysC and UcyC showed significant negative correlations with eGFR (a, b) and significant positive correlations with UACR (c, d). Abbreviations: CysC, serum cystatin C; eGFR, estimated glomerular filtration rate; UACR, urine albumin‐to‐creatinine ratio; UcyC, urinary cystatin C.

**Figure:**
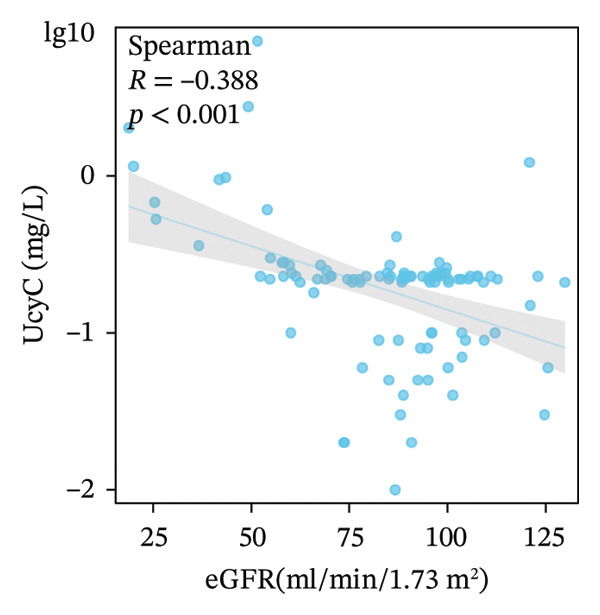
(a)

**Figure:**
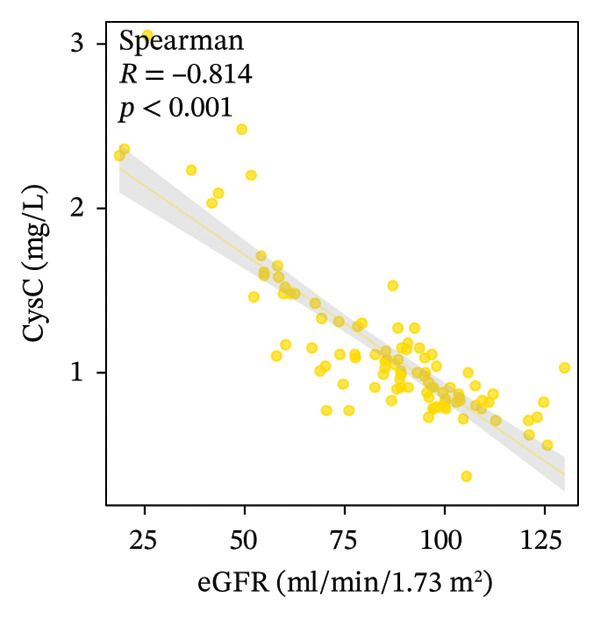
(b)

**Figure:**
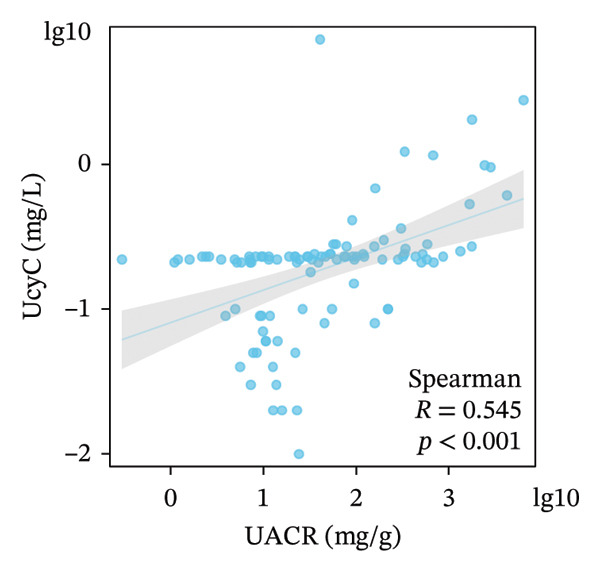
(c)

**Figure:**
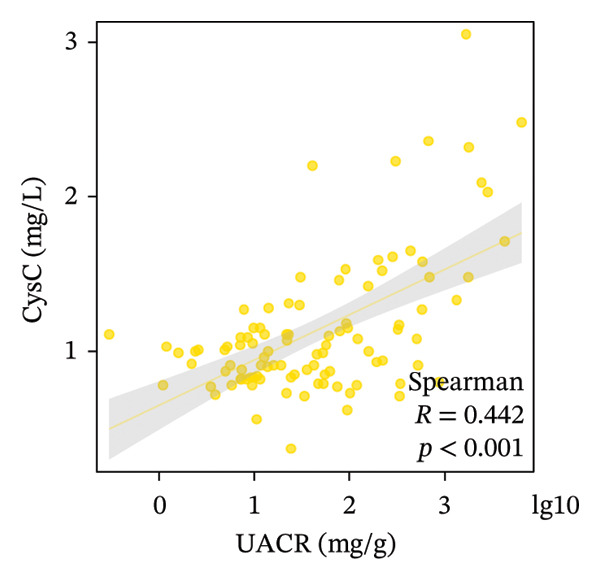
(d)

### 3.4. Diagnostic Value of Biomarkers for DKD Evaluated Through ROC Curve Analysis

To further assess the diagnostic utility of UcyC and CysC across different stages of DKD, we performed ROC curve analyses. In differentiating DKD among patients with T2DM, UcyC showed robust diagnostic performance, with an AUC of 0.830 (95% CI: 0.755–0.905), substantially outperforming CysC, which yielded an AUC of 0.691 (95% CI: 0.583–0.798) (Figures 4(a) and 4(b)). Notably, UcyC and CysC achieved perfect specificity and a PPV of 100.00%. However, UcyC exhibited higher sensitivity (50.00% vs. 41.18%), higher NPV (64.00% vs. 61.54%), and slightly greater overall accuracy (73.53% vs. 69.70%) than CysC. For EDKD diagnosis, UcyC showed modest discriminatory power (AUC = 0.618; 95% CI: 0.508–0.728), exceeding that of CysC (AUC = 0.507; 95% CI: 0.380–0.634) (Figures 4(c) and 4(d)). In this setting, UcyC retained perfect sensitivity and NPV (100.00% each) but exhibited low specificity (21.74%). As DKD progressed to CDKD, UcyC exhibited stronger discriminatory power than CysC, with AUCs of 0.844 and 0.794, respectively. UcyC achieved high specificity (85.19%), high NPV (92.00%), and high overall accuracy (82.35%), with moderate sensitivity (71.43%) and PPV (55.56%) (Figures 4(e) and 4(f)). Meanwhile, for differentiating EDKD from CDKD, both biomarkers showed promising and comparable diagnostic performance, with AUCs of 0.737 and 0.745, respectively (Figures 4(g) and 4(h)). UcyC maintained acceptable specificity (75.76%) (Supporting Table 2).

FIGURE 4ROC curve analysis showing the diagnostic value of UcyC and CysC across different stages of DKD. (a, b) Diagnostic performance of UcyC and CysC in detecting DKD in patients with T2DM. (c, d) Diagnostic performance of UcyC and CysC in detecting EDKD in patients with T2DM. (e, f) Diagnostic performance of UcyC and CysC in detecting CDKD in patients with T2DM. (g, h) Discriminatory power of UcyC and CysC in distinguishing disease severity among patients with DKD. Abbreviations: CDKD, clinical diabetic kidney disease; CysC, serum cystatin C; DKD, diabetic kidney disease; EDKD, early diabetic kidney disease; T2DM, Type 2 diabetes mellitus; T2DM without KD, T2DM without kidney disease; UcyC, urinary cystatin C.

**Figure:**
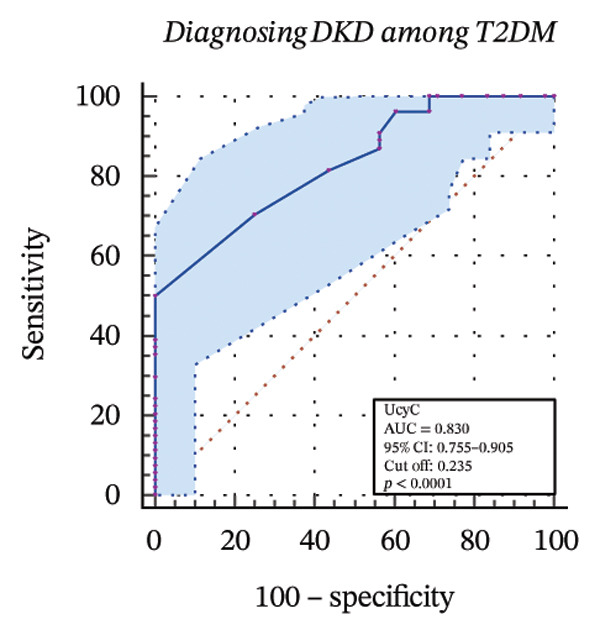
(a)

**Figure:**
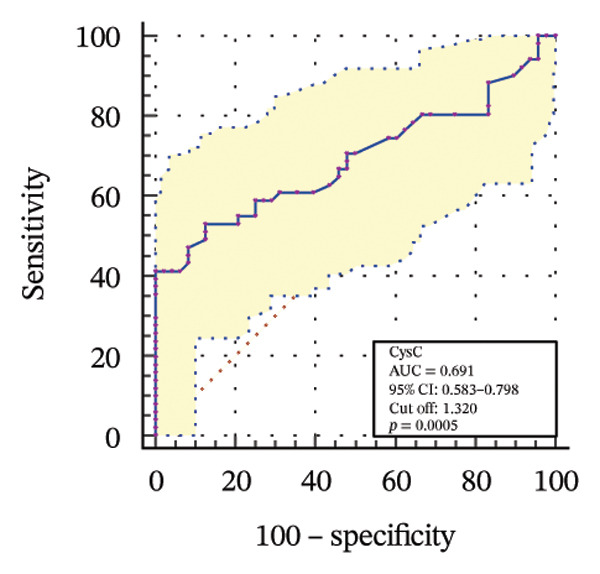
(b)

**Figure:**
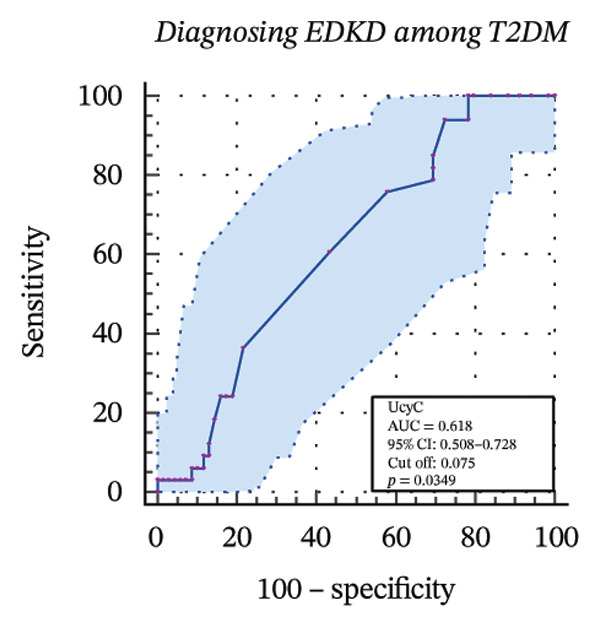
(c)

**Figure:**
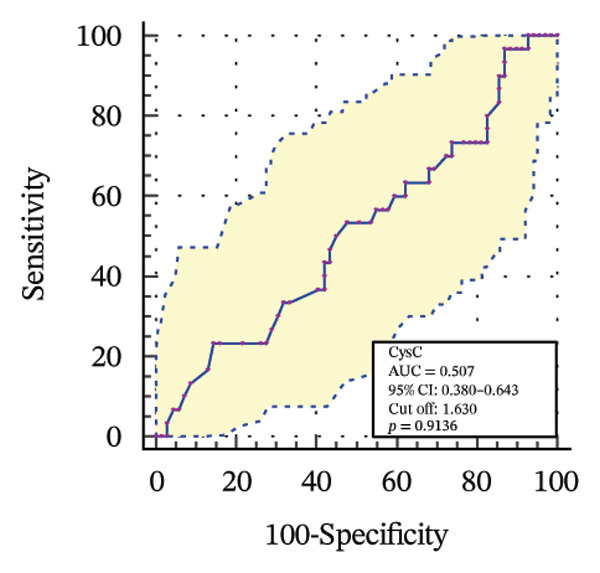
(d)

**Figure:**
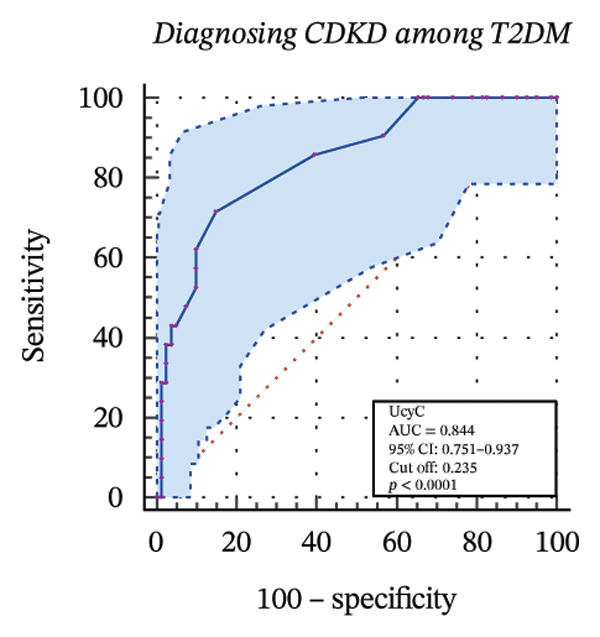
(e)

**Figure:**
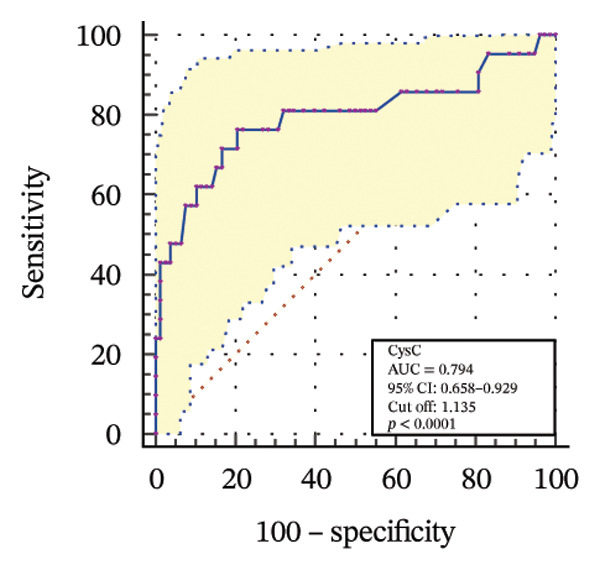
(f)

**Figure:**
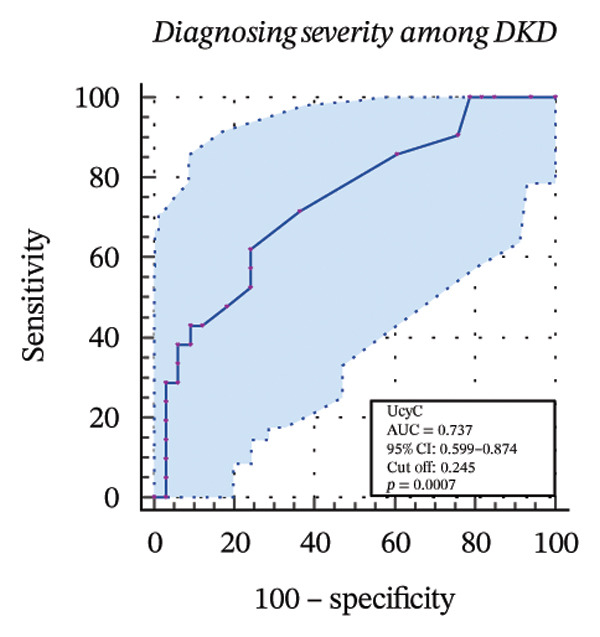
(g)

**Figure:**
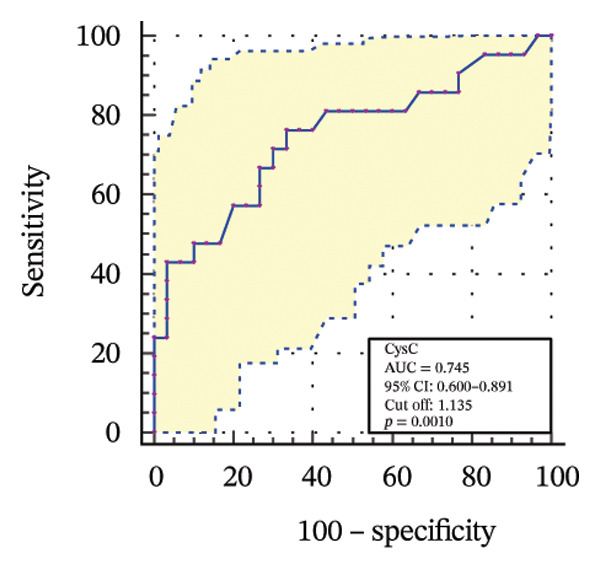
(h)

Collectively, these findings support UcyC as a valuable and specific biomarker for the early detection and clinical staging of DKD. Its comparatively superior sensitivity and NPV highlight its utility in differential diagnosis across disease stages and in guiding clinical decision‐making.

### 3.5. Association of Clinical Covariates With UcyC: Groupwise and Spearman Analyses

UcyC concentrations remained largely consistent across clinical subgroups in the diabetic cohort. As presented in Table 3, UcyC levels did not differ significantly according to gender, smoking status, alcohol consumption, or treatment regimens, including ACEI/ARB therapy, insulin use, oral hypoglycemic agents, or statin therapy. Hypertension was marginally associated with UcyC levels (*p* = 0.086). Conversely, the presence of microvascular complications—specifically diabetic retinopathy or neuropathy—was associated with significantly higher UcyC levels (0.23 vs. 0.22 mg/L; *p* = 0.031) (Figure 5(a)). Thus, among the categorical clinical variables examined, microvascular disease was the only factor showing a statistically significant association with UcyC levels in the groupwise analyses.

**TABLE 3:** Comparison of urinary cystatin C levels across clinical characteristic subgroups.

Characteristic	Subgroup	*n* (%)	UcyC (mg/L)	*p* value
Gender				0.903
	Male	55 (53.9)	0.22 (0.10, 0.24)	
	Female	77 (46.1)	0.22 (0.21, 0.24)	
Smoking status				0.457
	Never smoker	82 (80.4)	0.22 (0.19, 0.23)	
	Current/former smoker	20 (19.6)	0.23 (0.10, 0.24)	
Alcohol consumption				0.156
	Non‐drinker	77 (75.5)	0.22 (0.10, 0.23)	
	Drinker	25 (24.5)	0.23 (0.21, 0.25)	
Hypertension				0.086
	Absent	40 (39.2)	0.22 (0.10, 0.23)	
	Present	62 (60.8)	0.23 (0.21, 0.26)	
ACEI/ARB use				0.243
	No	81 (79.4)	0.23 (0.18, 0.24)	
	Yes	21 (20.6)	0.22 (0.08, 0.23)	
Insulin use				0.102
	No	71 (69.6)	0.22 (0.10, 0.23)	
	Yes	31 (30.4)	0.23 (0.22, 0.26)	
Non‐insulin glucose‐lowering agents			0.571	
	No	46 (45.1)	0.23 (0.10, 0.24)	
	Yes	56 (54.9)	0.22 (0.21, 0.23)	
Statins				0.838
	No	90 (88.2)	0.23 (0.10, 0.24)	
	Yes	12 (11.8)	0.22 (0.21, 0.24)	
Microvascular complications				**0.031**
	No	57 (55.9)	0.22 (0.09, 0.23)	
	Yes	45 (44.1)	0.23 (0.22, 0.25)	

*Note:* Data are presented as *n* (%) or median (IQR). Differences in continuous variables were assessed with the Mann–Whitney *U* test. Statistically significant differences (*p* < 0.05) are shown in bold. Non‐insulin glucose‐lowering agents include sodium‐glucose cotransporter 2 inhibitors (SGLT‐2i), glucagon‐like peptide‐1 receptor agonists (GLP‐1 RAs), thiazolidinediones (TZDs), insulin secretagogues, metformin, and α‐glucosidase inhibitors. Microvascular complications comprise a composite of diabetic retinopathy and neuropathy.

Abbreviations: ACEI, angiotensin‐converting enzyme inhibitors; ARB, angiotensin II receptor blockers; IQR, interquartile range.

FIGURE 5Primary correlates of UcyC excretion. (a) UcyC levels were significantly higher in participants with microvascular complications, specifically clinically diagnosed diabetic retinopathy or neuropathy (Microvasc Comp; *p* = 0.031, Mann–Whitney *U* test). Boxes represent the interquartile range, and horizontal lines indicate the median. (b–d) Scatterplots showing significant associations between UcyC and specific lipoprotein components: (b) inverse correlation with ApoE (*ρ* = −0.354, *p* < 0.001), (c) positive correlation with Lp(a) (*ρ* = 0.313, *p* = 0.002), and (d) inverse correlation with ApoA1 (*ρ* = −0.208, *p* = 0.043). Solid lines represent linear regression fits. UcyC, Lp(a), and ApoA1 were natural log–transformed for analysis. Abbreviations: ApoA1, apolipoprotein A1; ApoE, apolipoprotein E; Lp(a), lipoprotein(a); UcyC, urine cystatin C.

**Figure:**
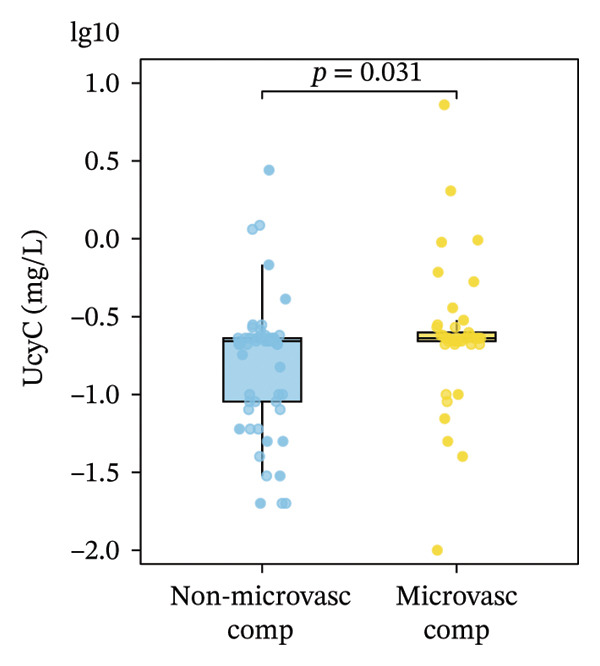
(a)

**Figure:**
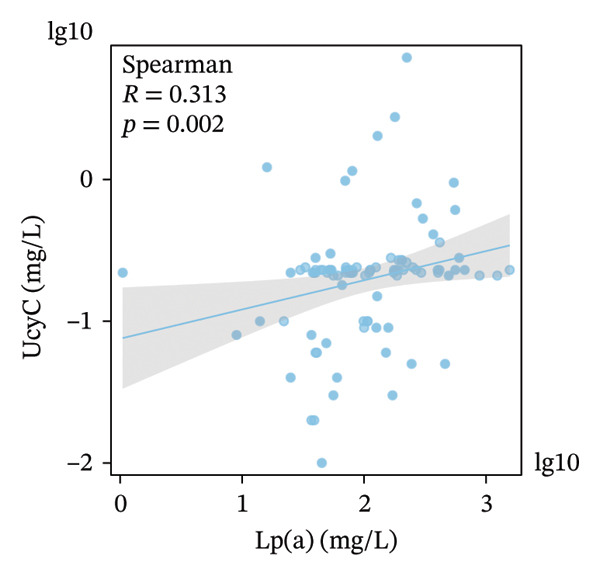
(b)

**Figure:**
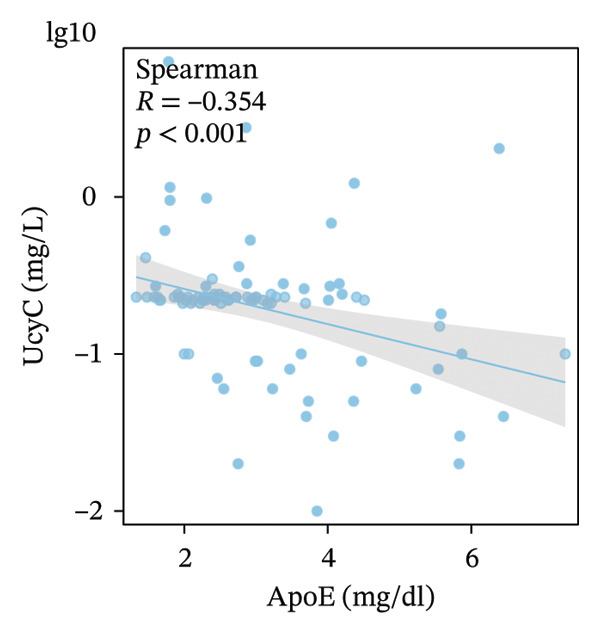
(c)

**Figure:**
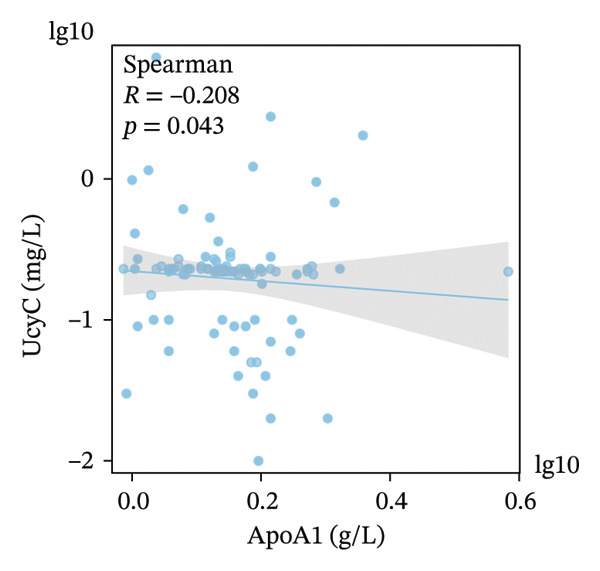
(d)

Beyond clinical phenotypes, correlation analyses revealed distinct relationships between UcyC and continuous metabolic parameters (Supporting Table 3). UcyC was not associated with glycemic indices (FBG, *p* = 0.415; HbA1c, *p* = 0.538) or with most conventional lipids, including TG (*p* = 0.592), TC (*p* = 0.402), and LDL‐C (*p* = 0.339). However, specific lipoprotein components correlated with UcyC:ApoE showed a moderate inverse correlation (*ρ* = −0.354, *p* < 0.001) (Figure 5(c)), Lp(a) was positively correlated (*ρ* = 0.313, *p* = 0.002) (Figure 5(b)), and ApoA1 showed a modest negative correlation (*ρ* = −0.208, *p* = 0.043) (Figure 5(d)). These lipid‐related associations, which remain understudied, warrant further mechanistic evaluation.

Synthesizing these findings, UcyC excretion exhibits three distinct characteristics: (1) its selective elevation in the context of microvascular complications suggests specificity for microangiopathic pathways rather than reflecting generalized metabolic perturbations; (2) the observed correlations with ApoE, Lp(a), and ApoA1 suggest potential lipoprotein‐mediated mechanisms influencing tubular handling of UcyC; (3) its consistent stability across demographic factors and routine pharmacotherapies underscores its potential as a reliable biomarker. The physiological significance of these associations—including the involvement of ApoE in renal protein reabsorption and the proatherogenic properties of Lp(a)—warrants targeted exploration in preclinical and clinical models of diabetic renal disease.

### 3.6. Independent Risk Factors for DKD: Univariate and Multivariate Analyses

Factors associated with DKD were assessed using univariate and multivariate logistic regressions. Variables with suggestive associations in univariate analysis (*p* < 0.10) (Supporting Table 4) were then entered, together with key covariates (age, sex, BMI), into the multivariate model (Table 4). After covariate adjustment, two factors emerged as statistically significant independent predictors of DKD. FBG was positively associated with DKD risk (adjusted odds ratio [aOR] = 1.343; 95% CI: 1.013–1.782, *p* = 0.041). Most notably, log10‐transformed UcyC (log10UcyC) showed a strong independent predictive value (aOR = 352.223; 95% CI: 3.567–34783.500, *p* = 0.012). Associations observed in univariate analysis for hypertension, CysC, ALP, and Neut# were no longer statistically significant after multivariate adjustment (all *p* > 0.05). Similarly, the marginal univariate relationships for SBP, DBP, and SGLT‐2i use did not persist in the adjusted model (all *p* > 0.05).

**TABLE 4:** Univariate and multivariate analysis of risk factors for diabetic kidney disease.

Characteristics	Univariate analysis		Multivariate analysis	
	OR (95% CI)	*p* value	aOR^a^ (95% CI)	*p* value
*Hypertension*				
No	Reference		Reference	
Yes	3.377 (1.469–7.762)	**0.004**	1.983 (0.362–10.858)	0.430
SBP	1.021 (1.000–1.043)	0.052	0.970 (0.917–1.027)	0.295
DBP	1.041 (0.998–1.085)	0.060	1.108 (0.995–1.238)	0.062

*SGLT-2i*				
No	Reference		Reference	
Yes	7.000 (0.829–59.136)	0.074	3.743 (0.178–78.749)	0.396
FCP	1.406 (0.954–2.071)	0.085	1.505 (0.595–3.810)	0.388
FBG	1.164 (1.038–1.305)	**0.009**	1.343 (1.013–1.782)	**0.041**
2hPBG	1.063 (0.991–1.140)	0.090	0.932 (0.795–1.094)	0.390
ALB	0.929 (0.853–1.012)	0.092	1.208 (0.953–1.533)	0.119
TBIL	0.920 (0.850–0.997)	**0.041**	0.890 (0.653–1.202)	0.459
DBIL	0.746 (0.543–1.024)	0.070	0.884 (0.208–3.759)	0.868
log10AST	0.089 (0.011–0.725)	**0.024**	0.009 (0.000–1.783)	0.081
ALP	1.018 (1.003–1.033)	**0.018**	1.016 (0.979–1.055)	0.398
CYCS	18.382 (3.537–95.527)	**< 0.001**	5.475 (0.086–349.634)	0.423
WBC	1.251 (0.979–1.600)	0.073	0.562 (0.150–2.106)	0.392
Neut#	1.385 (1.039–1.846)	**0.026**	2.327 (0.525–10.113)	0.266
Hb	0.980 (0.958–1.002)	0.072	0.946 (0.880–1.018)	0.136
log10UcyC	92.858 (9.495–908.158)	**< 0.001**	352.223 (3.567–34783.5001)	**0.012**

Abbreviations: OR, odds ratio; CI, confidence interval; SBP, systolic blood pressure; DBP, diastolic blood pressure; SGLT‐2i, sodium–glucose cotransporter 2 inhibitors; FCP, fasting C‐peptide; FBG, fasting blood glucose; 2hPBG, 2‐h postprandial blood glucose; ALB, albumin; TBIL, total bilirubin; DBIL, direct bilirubin; AST, aspartate aminotransferase; ALP, alkaline phosphatase; CysC, serum cystatin C; UcyC, urine cystatin C; WBC, white blood cell count; Neut#, neutrophil count; Hb, hemoglobin.

^a^Adjusted for age, sex, and BMI. log10 transformation was applied to UcyC and AST to address model nonconvergence due to complete separation. Statistically significant differences (*p* < 0.05) are shown in bold.

The robustness of log10UcyC as an independent risk factor for DKD was confirmed using sequential multivariate logistic regression models incorporating progressively comprehensive covariate sets (Table 5). In Model 1, adjusted for demographic and clinical parameters (sex, age, BMI, SBP, DBP, diabetes duration, and smoking), log10UcyC was strongly associated with DKD (OR = 450.532; 95% CI: 18.710–10848.925, *p* < 0.001). Model 2, which accounted for medication use and complication history, yielded a similar association (OR = 518.544, 95% CI: 17.107–15718.315; *p* < 0.001). In Model 3, inclusion of FBG did not attenuate the association (OR = 332.641; 95% CI: 10.891–10159.745; *p* = 0.001). Finally, Model 4, which additionally incorporated ApoA1, ApoE, and Lp(a), confirmed log10UcyC as a highly significant predictor (OR = 1617.907; 95% CI: 12.973–201769.226; *p* = 0.003). Across all models, predictive performance, assessed using AUC, remained consistently high, reaching 0.886 in Model 4, with sensitivity, negative predictive value, and overall accuracy registering at 94%, 91.43%, and 83.16%, respectively (Supporting Table 5).

**TABLE 5:** Independent association of log10UcyC with DKD across sequential multivariate models.

Model	Adjustment variables	log10UcyC OR (95% CI)	*p* value	AUC
Model 1	Sex, age, BMI, SBP, DBP, diabetes duration, smoking status	450.532 (18.710–10848.925)	**< 0.001**	0.837
Model 2	Model 1 + medication use, micro‐ or macrovascular complications	518.544 (17.107–15718.315)	**< 0.001**	0.863
Model 3	Model 2 + FBG	332.641 (10.891–10159.745)	**0.001**	0.881
Model 4	Model 3 + ApoA1, ApoE, Lp (a)	1617.907 (12.973–201769.226)	**0.003**	0.886

*Note:* Log transformation resolved complete separation at raw concentration cutpoints. Medications include ACEI, ARB, SGLT‐2i, GLP‐1 RAs, TZDs, insulin secretagogues, insulin, metformin, or statins. Micro‐ or macrovascular complications include diabetic peripheral arterial disease, diabetic peripheral neuropathy, or diabetic retinopathy. Statistically significant differences (*p* < 0.05) are shown in bold.

Abbreviations: ACEI, angiotensin‐converting enzyme inhibitors; ApoA1, apolipoprotein A1; ApoE, apolipoprotein E; ARB, angiotensin II receptor blockers; AUC, area under the curve; BMI, body mass index; CI, confidence interval; DBP, diastolic blood pressure; FBG, fasting blood glucose; GLP‐1 RAs, glucagon‐like peptide‐1 receptor agonists; Lp(a), lipoprotein(a); OR, odds ratio; SBP, systolic blood pressure; SGLT‐2i, sodium‐glucose cotransporter 2 inhibitors; TZDs, thiazolidinediones.

Collectively, these stratified analyses show that, following log10 transformation, elevated UcyC is independently associated with DKD risk and has stronger predictive value than conventional risk factors such as hypertension or dyslipidemia in fully adjusted models. Fasting hyperglycemia remains an independent, modifiable risk factor. The robust and consistent association of log10UcyC supports its utility as a biomarker for DKD risk stratification.

## 4. Discussion

DKD remains a major clinical challenge in the management of T2DM, progressing insidiously from early glomerular and tubular alterations to ESRD, often before timely intervention can be implemented. A critical barrier to improving patient outcomes lies in the limited ability of conventional surveillance tools—specifically, the UACR and eGFR—which typically reflect renal injury only after significant functional impairment has occurred [17]. Although UACR is widely used as a DKD biomarker, its diagnostic performance is susceptible to extrinsic factors, including posture, physical activity, and acute infections, which can introduce substantial variability [4, 18]. Furthermore, UACR lacks specificity for DKD, as its elevations may also be associated with comorbidities, such as cardiovascular diseases [19]. In this study, we systematically assessed the clinical utility of multiple biomarkers across distinct stages of DKD, with a particular emphasis on UcyC and CysC. Notably, UcyC showed a strong correlation with declining renal function and exhibited considerable promise as an early, sensitive indicator of DKD.

In this study, patients with DKD exhibited a significantly higher prevalence of hypertension and significantly elevated levels of FBG, ALP, and CREA compared with individuals with T2DM without KD, whereas ALB levels were significantly lower (all *p* < 0.05). These findings are consistent with those reported by Men et al., who identified associations between glucose metabolism indices, renal function indices, bone metabolism–related markers, and DKD [20]. Similarly, in agreement with the findings of Li et al., urea levels were significantly increased in patients with DKD [21]. Additionally, CysC levels were significantly elevated in DKD, consistent with Guo et al.’s observations regarding plasma CysC in diabetic nephropathy [3].

Consistent with the existing literature, our findings provide robust evidence supporting the clinical utility of UcyC in the assessment and management of DKD. In our cohorts, UcyC concentrations increased progressively with disease severity, ranging from 0.21 mg/L in T2DM without KD to 0.23 mg/L in EDKD and 0.27 mg/L in CDKD. Similarly, Hassan et al. evaluated the utility of UcyC as an early biomarker of DKD in patients with T2DM and reported a stepwise increase in UcyC levels with worsening proteinuria, thereby reinforcing the reliability of our findings. In that study, UcyC was positively correlated with UACR (*ρ* = 0.441) and negatively correlated with eGFR (*ρ* = −0.286). These results align closely with our observations, showing a positive association between UcyC and UACR (*r* = 0.545) and a negative correlation with eGFR (*r* = −0.388) [22]. Conversely, a retrospective study by Zhang et al. involving 92 patients with T2DM reported a different pattern of UcyC distribution across disease stages [23], with values of 0.15 mg/L in non‐DKD, 0.27 mg/L in EDKD, and 1.00 mg/L in CDKD. Nevertheless, consistent with previous studies [24], our results indicate that the concurrent increase in UcyC and decline in eGFR may enhance the early detection of DKD. Furthermore, Sinna and Altaf reported that UcyC exhibits superior performance in disease staging compared with its serum counterpart in cohorts with CKD [25].

Notably, subgroup and correlation analyses revealed distinct characteristics of UcyC that further support its clinical utility. UcyC concentrations remained stable across subgroups stratified by sex, smoking status, alcohol consumption, and commonly used pharmacotherapies, including ACEI/ARB, insulin, oral hypoglycemic agents, and statins. These findings are consistent with those of Wiesli et al. [26]. Notably, the presence of microvascular complications, specifically diabetic retinopathy and/or neuropathy, was associated with modest but statistically significant elevations in UcyC levels (0.23 vs. 0.22 mg/L; *p* = 0.031). This finding supports the specificity of UcyC for microangiopathic pathways and is consistent with the established pathological relationship between renal and retinal microvascular injury in diabetes [27]. The correlations between UcyC and specific lipoproteins provide additional novel mechanistic insights. UcyC was inversely associated with ApoE (*ρ* = −0.354) and ApoA1 (*ρ* = −0.208) while showing a positive association with Lp(a) (*ρ* = 0.313). These relationships are broadly consistent with known patterns of lipoprotein dysregulation in DKD [28]. Given the established role of ApoE in renal protein reabsorption and the proatherogenic properties of Lp(a), these correlations suggest that lipoprotein‐related mechanisms may influence the tubular handling of UcyC. This interpretation is a hypothesis that is not previously reported in the DKD literature and warrants further investigation.

A key contribution of this study is the novel identification of log10UcyC as a strong, independent predictor of DKD. In multivariable regression analyses, log10UcyC remained statistically significant (aOR = 352.223, *p* = 0.012) and showed excellent discriminative performance after full covariate adjustment (AUC = 0.886 in Model 4). The predictive power of UcyC persisted after including ApoA1, ApoE, and Lp(a) in the model, indicating that UcyC captures pathogenic information not fully explained by lipoprotein dysregulation. However, the markedly wide CI associated with this OR indicates uncertainty in the effect size estimate, underscoring the need for larger‐scale studies using independent cohorts.

UcyC exhibits superior stability and specificity compared with conventional biomarkers, particularly in EDKD, where it demonstrates greater sensitivity to subtle renal changes [22]. This study highlights the significant role of UcyC in the detection and staging of DKD. ROC curve analysis revealed that UcyC (AUC = 0.830; 95% CI: 0.74–0.89) outperformed CysC (AUC = 0.691) in identifying DKD among patients with T2DM, with high specificity and PPV. In EDKD, UcyC showed moderate discriminative ability (AUC = 0.618) but achieved perfect sensitivity (100.00%) and NPV, supporting its utility as an exclusionary screening marker. As DKD progresses to CDKD, the diagnostic efficacy of UcyC improves further (AUC = 0.844). These findings align with previous evaluations of renal injury biomarkers. For example, urinary neutrophil gelatinase–associated lipocalin (NGAL) has been reported to yield AUC values ranging from 0.84 to 0.90 for DKD detection, comparable to those observed for UcyC; however, UcyC demonstrates higher specificity (100% vs. 81%) [29]. Previous studies have also highlighted the diagnostic value of combining multiple biomarkers. In this context, Li et al. reported that the combined detection of multiple urinary tract biomarkers in early diabetic nephropathy achieved an AUC of 0.812 [30]. Conversely, our findings indicate that UcyC alone may offer strong diagnostic performance, particularly due to its high NPV, which may facilitate more streamlined clinical screening processes. Collectively, these results indicate that UcyC is a promising biomarker for the early detection and staging of DKD, with diagnostic performance comparable to or surpassing that of established markers, thereby supporting its potential to improve DKD assessment.

Several limitations merit consideration when interpreting our findings. First, although the study cohort (*n* = 102) was well characterized clinically, it did not include several key DKD subgroups—specifically, individuals with glomerular hyperfiltration (defined as eGFR > 125 mL/min/1.73 m^2^) and those with a nonalbuminuric DKD (NADKD) phenotype. This underrepresentation is clinically relevant, given that glomerular hyperfiltration reflects an early pathophysiological stage of DKD and NADKD accounts for a substantial proportion of diabetic renal involvement in clinical practice, estimated at 20%–40% of all DKD cases [31]. Notably, nondiabetic KD (NDKD) was also not represented in the cohort—a limitation inherently attributable to diagnostic constraints. Definitive diagnosis of NDKD requires histopathological confirmation through renal biopsy, and the relatively small sample size precluded the accumulation of sufficient NDKD cases that could meet statistical or clinical diagnostic thresholds. Consequently, the generalizability of these findings to these underrepresented populations is limited, and the utility of UcyC for detecting prealbuminuric or very early renal injury preceding microalbuminuria could not be adequately evaluated. Moreover, this was a single‐center, observational study conducted in a highly homogeneous Han Chinese population, and selection bias cannot be entirely excluded. Therefore, the cohort may not reflect the demographic, clinical, or genetic heterogeneity of broader populations with diabetes worldwide. External validation in larger, multicenter cohorts with diverse ethnic and geographic backgrounds is therefore necessary to confirm the robustness and generalizability of the diagnostic performance of UcyC. Another important limitation is the absence of histopathological correlations. While significant correlations were observed between UcyC and established biomarkers of renal function, such as UACR and eGFR, the lack of renal biopsy data prevented definitive conclusions concerning the direct association between UcyC levels and specific patterns of renal tissue injury, such as tubular atrophy, interstitial fibrosis, or glomerular lesions. Without such pathological corroboration, the precise anatomical and molecular mechanisms underlying increased UcyC excretion in DKD pathogenesis remain speculative. Finally, multivariate logistic regression analyses yielded an extremely broad CI for the OR associated with UcyC (data not shown), indicating statistical instability. This imprecision can be attributed to the relatively small sample size and the potential for unmeasured confounding factors. Such instability underscores the need for larger‐scale replication studies with adequate statistical power to more accurately quantify the independent prognostic value of UcyC for DKD onset and progression and validate its optimal cutoff values across diverse clinical settings.

Despite these limitations, UcyC retains considerable clinical value that warrants further evaluation. UcyC offers several practical advantages as a urine‐based biomarker. Sampling is noninvasive, collection is straightforward, and the results are largely insensitive to diurnal fluctuations or the timing of sample collection. Moreover, UcyU can be reliably detected using simple, rapid methods—such as enzyme‐linked immunosorbent assays or point‐of‐care testing [32, 33]. These attributes collectively render UcyC particularly suitable for large‐scale population screening and widespread use in resource‐constrained primary health care settings, where access to sophisticated laboratory infrastructure or invasive procedures is often limited. Importantly, these attributes distinguish UcyC from conventional renal biomarkers, including CysC, CREA, and eGFR. These indicators rely on invasive venous blood sampling, which not only causes physical distress for patients but also introduces the risks of sampling errors, hemolysis, and delays in specimen transport and processing—all of which can undermine the reliability of results. The noninvasive nature of UcyC is particularly advantageous for high‐risk populations requiring long‐term monitoring, such as patients with T2DM, older adults, or individuals with comorbidities that limit tolerance to repeated blood draws. In such populations, routine UcyC monitoring may enable early detection of subtle renal dysfunction—often preceding overt microalbuminuria or a decline in eGFR. This facilitates the timely initiation of renoprotective interventions, including ACEI/ARB, optimized glycemic, and lipid control. Ultimately, this proactive approach may contribute to improving clinical outcomes by slowing or halting the progression of DKD.

To address the limitations and fully realize the clinical potential of UcyC, future research should prioritize three interconnected objectives. First, integrating UcyC with other established tubular injury biomarkers represents a particularly promising strategy. Candidate markers include NGAL, kidney injury molecule‐1, and liver‐type fatty acid–binding protein, which may serve as the basis for composite diagnostic panels. Such multimarker approaches may overcome the limitations of single‐biomarker strategies, enhancing diagnostic sensitivity for early renal injury and improving the ability to distinguish DKD from NDKD—which remains an unmet clinical need highlighted in this study. Second, longitudinal cohort studies are essential to systematically validate UcyC dynamics across the entire pathophysiological spectrum of DKD, from glomerular hyperfiltration and prealbuminuric stages to overt clinical nephropathy. Such studies should examine how UcyC kinetics align with established renal biomarkers and histopathological progression, thereby clarifying its utility for monitoring disease trajectory and predicting long‐term renal outcomes, such as ≥ 30% eGFR decline and progression to ESRD. Third, large‐scale prospective studies in underrepresented high‐risk cohorts are critical. These cohorts include patients with glomerular hyperfiltration, NADKD, and comorbid cardiovascular disease, where early intervention may yield substantial clinical benefits. Conventional biomarkers often fail to detect subtle renal impairment in these groups, highlighting the potential value of UcyC. Finally, exploring the underlying molecular mechanisms linking UcyC excretion to tubular dysfunction may provide valuable insights into the pathogenesis of DKD and reveal therapeutic targets that extend beyond biomarker utility.

## 5. Conclusion

The findings of this study provide strong evidence that UcyC is a promising biomarker for the early detection and severity stratification of DKD in patients with T2DM. Compared with conventional biomarkers, UcyC shows superior diagnostic efficacy in detecting early renal injury and reliably reflects functional changes across progressive stages of DKD. Its high specificity, diagnostic accuracy, and noninvasive nature underscore its suitability as a screening tool for early kidney damage among vulnerable populations, offering new approaches for the timely diagnosis and personalized management of DKD.

## Funding

The study was supported by the National Key Research and Development Program of China (Project No. 2019YFF0216502).

## Ethics Statement

This study was approved by the Ethics Committee of The People’s Hospital of Chizhou (Approval No. 2021‐KY‐06). Owing to the rapid emergence of this infectious disease, written informed consent was waived.

## Conflicts of Interest

The authors declare no conflicts of interest.

## Supporting Information

Supporting Table 1: Baseline characteristics and laboratory indicators across diabetic kidney disease stages: T2DM without KD, EDKD, and CDKD.

Supporting Table 2: Comparison of the diagnostic performance of UcyC and CysC in different stages of diabetic kidney disease.

Supporting Table 3: Correlation analysis between urinary cystatin C and continuous clinical variables.

Supporting Table 4: Univariate regression analysis of clinical and laboratory characteristics in DKD patients.

Supporting Table 5: Sensitivity analysis for the association between log‐transformed urinary cystatin C and diabetic kidney disease.

## Supporting information


**Supporting Information** Additional supporting information can be found online in the Supporting Information section.

## Data Availability

The data that support the findings of this study are available from the corresponding author upon reasonable request.
