# Supplementary material for: Clinical Utility of Urinary Cystatin C in Early Screening and Staging of Diabetic Kidney Disease in Type 2 Diabetes
Source: Int J Endocrinol. 2026 Apr 22;2026:8881466. doi: 10.1155/ije/8881466 (PMC13100891; doi:10.1155/ije/8881466)
Supplement: Supplementary file 1 — Supporting Information Additional supporting information can be found online in the Supporting Information section. [file IJE-2026-8881466-s001.docx]

**Supplementary Materials**

**Supplement to Manuscript:**

**Clinical Utility of Urinary Cystatin C in Early Screening and Staging of Diabetic Kidney Disease in Type 2 Diabetes**

**Supplementary Materials Contents:**

Supplementary Table 1. 2

Supplementary Table 2. 9

Supplementary Table 3. 10

Supplementary Table 4. 11

Supplementary Table 5. 18

**Supplementary Table 1. Baseline characteristics and laboratory indicators across diabetic kidney disease stages: T2DM without KD, EDKD, and CDKD.**

| **Indicators** | **T2DM without KD**  **(n=48)** | **EDKD**  **(n=33)** | **CDKD**  **(n=21)** | ***P_overall_*** | ***P_1_*** | ***P_2_*** | ***P_3_*** |
| --- | --- | --- | --- | --- | --- | --- | --- |
| **Demographic data** |  |  |  |  |  |  |  |
| Gender (male), n (%) | 27 (56.3) | 14 (42.4) | 14 (66.7) | 0.199 | NA | NA | NA |
| Age (year), mean ± SD | 59.17 ± 11.53 | 60.21 ± 11.37 | 64.00 ± 11.52 | 0.274 | NA | NA | NA |
| BMI (kg/m^2^), mean ± SD | 24.30 ± 3.53 | 24.03 ± 3.66 | 26.35 ± 3.83 | 0.053 | NA | NA | NA |
| SBP (mm/Hg), median (IQR) | 135 (120, 145) | 136 (127, 150) | 148 (130, 152) | 0.076 | NA | NA | NA |
| DBP (mm/Hg), median (IQR) | 80 (71, 85) | 82.09 ± 7.91 | 83.86 ± 11.71 | 0.470 | NA | NA | NA |
| Smoking (yes), n (%) | 7 (14.6) | 7 (21.2) | 6 (28.6) | 0.203 | NA | NA | NA |
| Alcohol (yes), n (%) | 10 (20.8) | 7 (21.2) | 8 (38.1) | 0.267 | NA | NA | NA |
| Diabetes duration (year), median (IQR) | 5.0 (0.5, 14.3) | 7.0 (3.0, 8.0) | 10.0 (8.0, 20.0) | **0.004** | 1.000 | **0.005** | **0.014** |
| Family history of diabetes mellitus (yes), n (%) | 10 (20.8) | 9 (27.3) | 3 (14.3) | 0.512 | NA | NA | NA |
| **Underlying disease (yes), n (%)** |  |  |  |  |  |  |  |
| Hypertension | 22 (45.8) | 23 (69.7) | 17 (81.0) | **0.01** | 0.084 | **0.016** | 1.000 |
| Cerebral infarction | 4 (8.3) | 4 (12.1) | 2 (9.5) | 0.856 | NA | NA | NA |
| Hyperlipidemia | 16 (33.3) | 8 (24.2) | 8 (38.1) | 0.521 | NA | NA | NA |
| History of HBV infection | 5 (10.4) | 0 (0.0) | 2 (9.5) | 0.168 | NA | NA | NA |
| History of cataract | 6 (12.5) | 4 (12.1) | 3 (14.3) | 0.815 | NA | NA | NA |
| **Macrovascular/microvascular diabetic complications (Yes), n (%)** | | | | | | | |
| Peripheral arterial disease | 28 (58.3) | 23 (69.7) | 16 (76.2) | 0.299 | NA | NA | NA |
| Diabetic peripheral neuropathy | 18 (37.5) | 13 (39.4) | 12 (57.1) | 0.292 | NA | NA | NA |
| Diabetic retinopathy | 0 (0.0) | 1 (3.0) | 6 (28.6) | **< 0.001** | 1.000 | **< 0.001** | **< 0.001** |
| **Clinical symptoms (yes), n (%)** |  |  |  |  |  |  |  |
| Xerostomia | 37 (77.1) | 27 (81.8) | 12 (57.1) | 0.109 | NA | NA | NA |
| Polydipsia | 36 (75.0) | 25 (75.8) | 11 (52.4) | 0.121 | NA | NA | NA |
| Polyuria | 32 (66.7) | 26 (78.8) | 17 (81.0) | 0.329 | NA | NA | NA |
| Unintended weight loss | 19 (39.6) | 14 (42.4) | 4 (19.0) | 0.177 | NA | NA | NA |
| Paresthesia | 15 (31.3) | 13 (39.4) | 10 (47.6) | 0.413 | NA | NA | NA |
| Visual impairment | 8 (16.7) | 3 (9.1) | 5 (23.8) | 0.345 | NA | NA | NA |
| Blurred vision | 19 (39.6) | 14 (42.4) | 13 (61.9) | 0.214 | NA | NA | NA |
| Lower extremity edema | 2 (4.2) | 0 (0.0) | 4 (19.0) | **0.011** | 1.000 | **0.043** | **0.011** |
| Skin pruritus | 2 (4.2) | 1 (3.0) | 0 (0.0) | 0.648 | NA | NA | NA |
| Chest discomfort | 2 (4.2) | 0 (0.0) | 3 (14.3) | 0.057 | NA | NA | NA |
| Fatigue | 12 (25.0) | 7 (21.2) | 7 (33.3) | 0.605 | NA | NA | NA |
| **Current medication status (yes), n (%)** |  |  |  |  |  |  |  |
| ACEI | 4 (8.3) | 0 (0.0) | 1 (4.8) | 0.238 | NA | NA | NA |
| ARB | 7 (14.6) | 6 (18.2) | 3 (14.3) | 0.894 | NA | NA | NA |
| SGLT-2i | 1 (2.1) | 3 (9.1) | 4 (19.0) | 0.052 | NA | **0.048** | NA |
| GLP-1 RAs | 0 (0.0) | 2 (6.1) | 0 (0.0) | 0.121 | NA | NA | NA |
| TZDs | 1 (2.1) | 3 (9.1) | 0 (0.0) | 0.167 | NA | NA | NA |
| Insulin secretagogues | 8 (16.7) | 7 (21.2) | 7 (33.3) | 0.307 | NA | NA | NA |
| Insulin (yes) | 13 (27.1) | 8 (24.2) | 10 (47.6) | 0.151 | NA | NA | NA |
| Metformin | 20 (41.7) | 11 (33.3) | 9 (42.9) | 0.705 | NA | NA | NA |
| α-Glucosidase inhibitors | 5 (10.4) | 2 (6.1) | 2 (9.5) | 0.793 | NA | NA | NA |
| Statins | 7 (14.6) | 2 (6.1) | 3 (14.3) | 0.473 | NA | NA | NA |
| **Glucose metabolic indicators** |  |  |  |  |  |  |  |
| FCP (ng/mL), median (IQR) | 1.46 (0.93, 2.05) | 1.65 (1.32, 2.69) | 2.17 (1.70, 3.08) | 0.023 | 0.960 | **0.018** | 0.219 |
| 2hPP-CP (ng/mL), median (IQR) | 3.24 (2.18, 5.32) | 3.25 (1.95, 4.41) | 3.90 (2.98, 5.21) | 0.546 | NA | NA | NA |
| FBG (mmol/L), median (IQR) | 8.78 (6.74, 11.28) | 10.70 (8.66, 15.63) | 10.17 (7.77, 13.63) | **0.047** | 0.053 | 0.407 | 1.000 |
| 2hPBG (mmol/L), mean ± SD | 15.79 ± 5.13 | 18.15 ± 5.67 | 17.46 ± 7.87 | 0.182 | NA | NA | NA |
| HbA_1c_ (%), median (IQR) | 9.40 (7.28, 10.50) | 10.0 (8.5, 12.0) | 8.6 (7.6, 11.0) | 0.235 | NA | NA | NA |
| **Hepatic function indicators** |  |  |  |  |  |  |  |
| TP (g/L), median (IQR) | 68.04 ± 6.90 | 71.32 ± 7.23 | 66.81 ± 7.71 | 0.050 | 0.113 | 0.792 | 0.069 |
| ALB (g/L), mean ± SD | 41.21 ± 3.92 | 41.13 ± 4.62 | 37.15 ± 5.84 | **0.002** | 0.997 | **0.003** | **0.007** |
| TBIL (μmol/L), median (IQR) | 12.65 (11.05, 16.70) | 10.90 (9.40, 13.30) | 10.30 (8.90, 12.60) | **0.019** | 0.097 | **0.042** | 1.000 |
| DBIL (μmol/L), median (IQR) | 3.35 (2.60, 4.20) | 2.80 (2.60, 3.50) | 2.80 (2.20, 4.10) | 0.193 | NA | NA | NA |
| AST (U/L), median (IQR) | 19.00 (17.00, 30.00) | 19.50 (15.00, 23.00) | 19.00 (13.00, 25.50) | 0.104 | NA | NA | NA |
| ALT (U/L), median (IQR) | 19.50 (16.00, 34.25) | 19.00 (10.00, 27.00) | 17.00 (11.00, 30.00) | 0.222 | NA | NA | NA |
| GGT (U/L), median (IQR) | 21.00 (14.75, 29.25) | 26.00 (18.00, 48.00) | 32.00 (19.00, 83.00) | 0.106 | NA | NA | NA |
| ALP (U/L), median (IQR) | 77.00 (58.00, 98.00) | 93.50 (80.00, 106.50) | 91.00 (70.50, 101.75) | **0.009** | **0.009** | 0.237 | 1.000 |
| **Kidney function indicators** |  |  |  |  |  |  |  |
| UREA (mmol/L), median (IQR) | 5.28 (4.71, 6.54) | 6.23 (4.97, 6.98) | 7.25 (5.77, 9.02) | **0.004** | 0.300 | **0.003** | 0.357 |
| CREA (umol/L), median (IQR) | 68.25 (55.70, 79.70) | 71.70 (61.50, 97.95) | 95.90 (82.20, 131.40) | **< 0.001** | 0.249 | **< 0.001** | 0.077 |
| UA (μmol/L), median (IQR) | 315.5 (237.5, 377.5) | 284.0 (240.0, 402.0) | 542.0 (300.0, 464.0) | 0.067 | NA | NA | NA |
| eGFR (ml/min/1.73m^2^), mean ± SD | 94.51 ± 14.11 | 81.83 ± 22.26 | 63.94 ± 28.72 | **< 0.001** | **0.015** | **< 0.001** | 0.052 |
| CysC (mg/L), median (IQR) | 0.92 (0.83, 1.08) | 1.00 (0.86, 1.36) | 1.48 (1.14, 2.09) | **< 0.001** | 0.501 | **< 0.001** | **0.013** |
| UCREA (mmol/L), median (IQR) | 7.60 (3.44, 12.36) | 7.80 (5.60, 11.30) | 8.50 (6.00, 11.10) | 0.684 | NA | NA | NA |
| UMALB (mg/L), median (IQR) | 11.05 (4.98, 16.45) | 74.50 (45.60, 107.80) | 1150.30 (365.10, 2223.10) | **< 0.001** | **< 0.001** | **< 0.001** | **0.005** |
| UACR (mg/g), median (IQR) | 9.55 (5.50, 14.05) | 77.60 (46.80, 121.90) | 674.70 (436.90, 1770.50) | **< 0.001** | **< 0.001** | **< 0.001** | **0.003** |
| UcyC (mg/L), median (IQR) | 0.21 (0.06, 0.22) | 0.23 (0.22, 0.24) | 0.27 (0.23, 0.95) | **< 0.001** | **< 0.001** | **< 0.001** | **0.048** |
| **Lipid metabolic indicators** |  |  |  |  |  |  |  |
| TG (mmol/L), median (IQR) | 1.31 (0.98, 2.21) | 1.44 (1.16, 2.54) | 1.63 (1.06, 2.33) | 0.511 | NA | NA | NA |
| TC (mmol/L), mean ± SD | 4.90 ± 1.25 | 4.82 ± 0.92 | 5.14 ± 1.41 | 0.624 | NA | NA | NA |
| HDL-C (mmol/L), median (IQR) | 1.22 (0.98, 1.54) | 1.12 (0.92, 1.27) | 1.04 (0.98, 1.62) | 0.253 | NA | NA | NA |
| LDL-C (mmol/L), median (IQR) | 2.50 (1.89, 3.01) | 2.59 (2.04, 3.13) | 2.18 (1.79, 3.29) | 0.816 | NA | NA | NA |
| ApoA1 (g/L), median (IQR) | 1.46 (1.22, 1.58) | 1.36 (1.18, 1.49) | 1.36 (1.32, 1.64) | 0.244 | NA | NA | NA |
| ApoB (g/L), mean ± SD | 0.89 ± 0.23 | 0.95 ± 0.22 | 0.98 ± 0.32 | 0.334 | NA | NA | NA |
| ApoE (mg/dl), median (IQR) | 2.97 (2.26, 3.70) | 2.58 (2.12, 3.47) | 2.76 (2.02, 3.67) | 0.872 | NA | NA | NA |
| NEFA (mmol/L), median (IQR) | 0.49 (0.31, 0.71) | 0.46 (0.34, 0.72) | 0.51 (0.36, 0.69) | 0.986 | NA | NA | NA |
| Lp(a) (mg/L), median (IQR) | 76 (41, 186) | 99(50, 175) | 202 (87, 417) | **0.024** | 1.000 | **0.031** | **0.043** |
| **Thyroid function indicators** |  |  |  |  |  |  |  |
| FT3 (pg/ml), mean ± SD | 2.50 ± 0.49 | 2.46 ± 0.49 | 2.45 ± 0.29 | 0.889 | NA | NA | NA |
| FT4 (ng/dl), median (IQR) | 0.98 (0.85, 1.07) | 1.00 (0.91, 1.11) | 0.96 (0.91, 1.04) | 0.587 | NA | NA | NA |
| TSH (μIU/ml), median (IQR) | 2.01 (1.41, 2.79) | 2.07 (1.57, 2.63) | 2.12 (1.88, 2.64) | 0.574 | NA | NA | NA |
| **Hematology indicators** |  |  |  |  |  |  |  |
| WBC (×10^9^/L), median (IQR) | 5.51 (4.71, 6.32) | 5.92 (4.98, 7.51) | 6.88 (5.07, 7.29) | 0.110 | NA | NA | NA |
| Neut# (×10^9^/L), median (IQR) | 3.01 (2.41, 3.94) | 3.77 (2.66, 4.87) | 3.60 (3.08, 4.73) | 0.063 | NA | NA | NA |
| Lym# (×109/L), mean ± SD | 1.82 ± 0.49 | 1.69 ± 0.63 | 1.84 ± 0.63 | 0.492 | NA | NA | NA |
| RBC (×1012/L), mean ± SD | 4.49 ± 0.53 | 4.35 ± 0.73 | 4.37 ± 0.69 | 0.549 | NA | NA | NA |
| Hb (g/L), median (IQR) | 134 (122, 145) | 131 (117, 147) | 126 (118, 138) | 0.325 | NA | NA | NA |
| PLT (×109/L), median (IQR) | 183 (154, 203) | 203 (138, 223) | 168 (106, 209) | 0.330 | NA | NA | NA |

Data are presented as mean ± SD, or median (IQR). Intergroup comparisons use Student’s *t*-test for normally distributed variables, the Mann-Whitney *U* test for non-normally distributed variables, and the Chi-square test or Fisher’s exact test for categorical variables, as appropriate. Intragroup comparisons employe the one-way ANOVA, or Kruskal-Wallis H test, followed by post-hoc Bonferroni-corrected pairwise comparisons: Poverall (overall difference among the three groups); P1 (Bonferroni-corrected for T2DM without KD vs EDKD); P2 (Bonferroni-corrected for T2DM without KD vs CDKD); P3 (Bonferroni-corrected for EDKD vs CDKD); NA, no statistically significant difference. Statistically significant differences (p < 0.05) are highlighted in bold typeface. IQR, Interquartile range;SD, standard deviation; BMI, body mass index; SBP, systolic blood pressure; DBP, diastolic blood pressure; ACEI, angiotensin-converting enzyme inhibitors; ARB, angiotensin II receptor blockers; SGLT-2i, sodium-glucose cotransporter 2 inhibitors; GLP-1 RAs, glucagon-like peptide-1 receptor agonists; TZDs, thiazolidinediones; FCP, fasting C-peptide; 2hPP-CP, 2-hour postprandial C-peptide; FBG, blood glucose; 2hPBG, 2-hour postprandial blood glucose; HbA1c, glycated hemoglobin A1c; TP, total protein; ALB, albumin; TBIL, total bilirubin; DBIL, direct bilirubin; AST, aspartate aminotransferase; ALT, alanine aminotransferase; GGT, gamma-glutamyl transferase; ALP, alkaline phosphatase; CREA, creatinine; eGFR, estimate glomerular filtration rate; UA, uric acid; CysC, serum cystatin C; UACR, urine microalbumin/creatinine ratio; UCREA, urine creatinine; UMALB, urine microalbumin； UcyC, urine cystatin C; TG, triglycerides; TC, total cholesterol; HDL-C, high-density lipoprotein cholesterol; LDL-C, low-density lipoprotein cholesterol; ApoA1, apolipoprotein A1; ApoB, apolipoprotein B; ApoE, apolipoprotein E; NEFA, non-esterified fatty acids; Lp(a), lipoprotein(a); FT3, free triiodothyronine; FT4, free thyroxine; TSH, thyroid-stimulating hormone; WBC, white blood cell count; Neut#, neutrophil count; Lym#, lymphocyte count; RBC, red blood cell count; Hb, hemoglobin; PLT, platelet count. UACR (mg/g) = [Urinary Albumin (mg/L) / Urinary Creatinine (mmol/L)] × 113 (where 113 is creatinine's molecular weight).

**Supplementary Table 2. Comparison of the diagnostic performance of UcyC and CysC in different stages of diabetic kidney disease.**

| **Indicators** | **Sensitivity (%)** | **Specificity (%)** | **Accuracy (%)** | **Positive Predictive Value (%)** | **Negative Predictive Value (%)** |
| --- | --- | --- | --- | --- | --- |
| ***Diagnosing DKD among T2DM patients*** | | | | | |
| Ucyc | 50.00 | 100 | 73.53 | 100 | 64.00 |
| CysC | 41.18 | 100 | 69.70 | 100 | 61.54 |
| ***Diagnosing EDKD among T2DM patients*** | | | | | |
| Ucyc | 100 | 21.74 | 47.06 | 37.93 | 100 |
| CysC | 96.67 | 13.04 | 38.38 | 32.58 | 90.00 |
| ***Diagnosing CDKD among T2DM patients*** | | | | | |
| Ucyc | 71.43 | 85.19 | 82.35 | 55.56 | 92.00 |
| CysC | 76.19 | 79.49 | 78.79 | 50.00 | 92.54 |
| ***Diagnosing severity among DKD patients*** | | | | | |
| Ucyc | 61.91 | 75.76 | 70.37 | 61.91 | 75.76 |
| CysC | 76.19 | 66.67 | 70.58 | 61.54 | 80.00 |

Diagnosing DKD among T2DM patients, negatives were just T2DM without KD patients (n = 48); Diagnosing EDKD among T2DM patients, negatives were T2DM without KD and CDKD patients (n = 69); Diagnosing CDKD among T2DM patients, negatives were T2DM without KD and EDKD patients (n = 81); Diagnosing severity among DKD patients, negatives were EDKD patients (n = 33).

**Supplementary Table 3. Correlation Analysis Between Urinary Cystatin C and Continuous Clinical Variables.**

| **Variable** | **n/N** | **Spearman's ρ** | ***p*-value** |
| --- | --- | --- | --- |
| **Demographic & Metabolic** |  |  |  |
| Age (year) | 102 / 102 | 0.187 | 0.060 |
| Diabetes duration (year) | 102 / 102 | 0.182 | 0.067 |
| BMI (kg/m²) | 102 / 102 | 0.033 | 0.746 |
| SBP (mmHg) | 102 / 102 | 0.070 | 0.482 |
| DBP (mmHg) | 102 / 102 | 0.016 | 0.877 |
| FBG (mmol/L) | 102 / 102 | 0.180 | 0.070 |
| HbA_1c_ (%) | 101 / 102 | 0.128 | 0.201 |
| **Lipid Profile** |  |  |  |
| TG (mmol/L) | 102 / 102 | 0.012 | 0.902 |
| TC (mmol/L) | 102 / 102 | –0.139 | 0.164 |
| HDL-C (mmol/L) | 102 / 102 | –0.187 | 0.060 |
| LDL-C (mmol/L) | 102 / 102 | –0.151 | 0.130 |
| ApoA1 (g/L) | 95 / 102 | –0.208 | **0.043** |
| ApoB (g/L) | 95 / 102 | 0.014 | 0.895 |
| ApoE (mg/dl) | 95 / 102 | –0.354 | **< 0.001** |
| NEFA (mmol/L) | 95 / 102 | 0.050 | 0.628 |
| Lp(a) (mg/L) | 95 / 102 | 0.313 | **0.002** |

Correlation coefficients (ρ) and P-values calculated using Spearman's rank-order correlation. Bold indicates statistical significance (P < 0.05). n/N, Valid sample size/total cohort size (N=102). BMI, body mass index; SBP, systolic blood pressure; DBP, diastolic blood pressure; FBG, fasting blood glucose; HbA_1c_, glycated hemoglobin A_1c_; TG, triglycerides; TC, total cholesterol; HDL-C, high-density lipoprotein cholesterol; LDL-C, low-density lipoprotein cholesterol; ApoA1, apolipoprotein A1; ApoB, apolipoprotein B; ApoE, apolipoprotein E; NEFA, non-esterified fatty acids; Lp(a), lipoprotein(a); eGFR, estimate glomerular filtration rate; UACR, urine microalbumin/creatinine ratio.

**Supplementary Table 4. Univariate gegression analysis of clinical and Laboratory characteristics in DKD Patients.**

| **Characteristics** | **Odds Ratio (95% CI)** | **P value** |
| --- | --- | --- |
| **Sex** |  |  |
| *Male* | Reference |  |
| *Female* | 1.194 (0.547–2.607) | 0.657 |
| **Age** | 1.020 (0.985–1.055) | 0.271 |
| **BMI** | 1.048 (0.942–1.166) | 0.391 |
| **SBP** | 1.021 (1.000–1.043) | **0.052** |
| **DBP** | 1.041 (0.998–1.085) | **0.060** |
| **Smoking** |  |  |
| *No* | Reference |  |
| *Yes* | 1.857 (0.673–5.128) | 0.232 |
| **Alcohol** |  |  |
| *No* | Reference |  |
| *Yes* | 1.462 (0.585–3.654) | 0.417 |
| **Diabetes duration** | 1.031 (0.974–1.091) | 0.299 |
| *No* | Reference |  |
| *Yes* | 1.086 (0.421–2.799) | 0.865 |
| **Hypertension** |  |  |
| *No* | Reference |  |
| *Yes* | 0.296 (0.129–0.681) | **0.004** |
| *No* | Reference |  |
| *Yes* | 1.375 (0.364–5.197) | 0.639 |
| **Hyperlipidemia** |  |  |
| *No* | Reference |  |
| *Yes* | 0.842 (0.364–1.946) | 0.688 |
| *No* | Reference |  |
| *Yes* | 0.331 (0.061–1.791) | 0.199 |
| *No* | Reference |  |
| *Yes* | 1.043 (0.325–3.350) | 0.944 |
| **Xerostomia** |  |  |
| *No* | Reference |  |
| *Yes* | 1.294 (0.527–3.178) | 0.574 |
| **Polydipsia** |  |  |
| *No* | Reference |  |
| *Yes* | 1.500 (0.632–3.560) | 0.358 |
| **Polyuria** |  |  |
| *No* | Reference |  |
| *Yes* | 1.955 (0.800–4.777) | 0.142 |
| *No* | Reference |  |
| *Yes* | 1.310 (0.583–2.943) | 0.513 |
| **Paresthesia** |  |  |
| *No* | Reference |  |
| *Yes* | 1.632 (0.723–3.686) | 0.238 |
| *No* | Reference |  |
| *Yes* | 0.870 (0.299–2.529) | 0.798 |
| **Blurred vision** |  |  |
| *No* | Reference |  |
| *Yes* | 1.526 (0.695–3.353) | 0.292 |
| *No* | Reference |  |
| *Yes* | 1.840 (0.322–10.525) | 0.493 |
| **Skin pruritus** |  |  |
| *No* | Reference |  |
| *Yes* | 0.434 (0.038–4.943) | 0.501 |
| **Chest discomfort** |  |  |
| *No* | Reference |  |
| *Yes* | 1.353 (0.216–8.459) | 0.747 |
| **Fatigue** |  |  |
| *No* | Reference |  |
| *Yes* | 0.952 (0.390–2.326) | 0.915 |
| **ACEI** |  |  |
| *No* | Reference |  |
| *Yes* | 0.208 (0.022–1.925) | 0.166 |
| **ARB** |  |  |
| *No* | Reference |  |
| *Yes* | 0.854 (0.291–2.500) | 0.773 |
| **SGLT-2i** |  |  |
| *No* | Reference |  |
| *Yes* | 7.000 (0.829–59.136) | **0.074** |
| **GLP-1 RAs** |  |  |
| *No* | Reference |  |
| *Yes* | 5314904.1785 (0.000–Inf) | 0.988 |
| **TZDs** |  |  |
| *No* | Reference |  |
| *Yes* | 2.765 (0.278–27.510) | 0.386 |
| **Insulin secretagogues** | | |
| *No* | Reference |  |
| *Yes* | 0.571 (0.216–1.512) | 0.260 |
| **Insulin** |  |  |
| *No* | Reference |  |
| *Yes* | 1.346 (0.574–3.155) | 0.494 |
| **Metformin** |  |  |
| *No* | Reference |  |
| *Yes* | 1.214 (0.548–2.693) | 0.633 |
| *No* | Reference |  |
| *Yes* | 0.688 (0.174–2.725) | 0.594 |
| **Statins** |  |  |
| *No* | Reference |  |
| *Yes* | 0.598 (0.176–2.025) | 0.408 |
| **FCP** | 1.406 (0.954–2.071) | **0.085** |
| **2hPP-CP** | 0.980 (0.851–1.128) | 0.774 |
| **FBG** | 1.164 (1.038–1.305) | **0.009** |
| **2hPBG** | 1.063 (0.991–1.140) | **0.090** |
| **HbA_1c_** | 1.088 (0.928–1.276) | 0.298 |
| **TP** | 1.030 (0.975–1.087) | 0.294 |
| **ALB** | 0.929 (0.853–1.012) | **0.092** |
| **TBIL** | 0.920 (0.850–0.997) | **0.041** |
| **DBIL** | 0.746 (0.543–1.024) | **0.070** |
| **AST** | 0.970 (0.938–1.003) | **0.075** |
| **ALT** | 0.986 (0.966–1.005) | 0.152 |
| **GGT** | 1.000 (0.995–1.005) | 0.872 |
| **ALP** | 1.018 (1.003–1.033) | **0.018** |
| **UA** | 1.003 (0.999–1.007) | 0.142 |
| **CysC** | 18.382 (3.537–95.527) | **< 0.001** |
| **log10UcyC** | 92.858 (9.495–908.158) | **< 0.001** |
| **TG** | 1.098 (0.878–1.373) | 0.412 |
| **TC** | 0.964 (0.870–1.068) | 0.480 |
| **HDL-C** | 0.617 (0.238–1.600) | 0.321 |
| **LDL-C** | 1.054 (0.649–1.712) | 0.831 |
| **APOA1** | 0.482 (0.140–1.660) | 0.248 |
| **APOB** | 3.418 (0.629–18.578) | 0.155 |
| **APOE** | 0.933 (0.679–1.280) | 0.666 |
| **NEFA** | 1.361 (0.473–3.913) | 0.568 |
| **LPa** | 1.000 (0.999–1.002) | 0.798 |
| **FT3** | 0.804 (0.336–1.923) | 0.624 |
| **FT4** | 1.693 (0.173–16.533) | 0.651 |
| **TSH** | 0.951 (0.675–1.340) | 0.773 |
| **WBC** | 1.251 (0.979–1.600) | **0.073** |
| **Neut#** | 1.385 (1.039–1.846) | **0.026** |
| **Lym#** | 0.791 (0.393–1.589) | 0.509 |
| **RBC** | 0.702 (0.373–1.321) | 0.272 |
| **HGB** | 0.980 (0.958–1.002) | **0.072** |
| **PLT** | 1.001 (0.997–1.005) | 0.673 |

log10 transformation of UcyC was applied to resolve complete separation-induced model non-convergence.OR, odds ratio; CI, confidence interval. BMI, body mass index (calculated as weight in kilograms divided by the square of height in meters); SBP, systolic blood pressure; DBP, diastolic blood pressure; ACEI, Angiotensin-converting enzyme inhibitors; ARB, Angiotensin II receptor blockers; SGLT-2i, Sodium-glucose cotransporter 2 inhibitors; GLP-1 RAs, Glucagon-like peptide-1 receptor agonists; TZDs, Thiazolidinediones. FCP, fasting C-peptide; 2hPP-CP, 2-hour postprandial C-peptide; FBG, fasting blood glucose; 2hPBG, 2-hour postprandial blood glucose; HbA_1c_, glycated hemoglobin A_1c_; TP, total protein; ALB, albumin; TBIL, total bilirubin; DBIL, direct bilirubin; AST, aspartate aminotransferase; ALT, alanine aminotransferase; GGT, gamma-glutamyl transferase; ALP, alkaline phosphatase; UA, uric acid; CysC, serum cystatin C; UcyC, urine cystatin C; TG, triglycerides; TC, total cholesterol; HDL-C, high-density lipoprotein cholesterol; LDL-C, low-density lipoprotein cholesterol; ApoA1, apolipoprotein A1; ApoB, apolipoprotein B; ApoE, apolipoprotein E; NEFA, non-esterified fatty acids; Lp(a), lipoprotein(a); FT3, free triiodothyronine; FT4, free thyroxine; TSH, thyroid-stimulating hormone; WBC, white blood cell count; Neut#, neutrophil count; Lym#, lymphocyte count; RBC, red blood cell count; Hb, hemoglobin; PLT, platelet count. Bolded P-values (P < 0.10) indicate variables that were considered statistically suggestive in univariate analysis and subsequently included in the multivariate regression model.

**Supplementary Table 5. Sensitivity Analysis for the Association Between Log-Transformed Urinary Cystatin C and Diabetic Kidney Disease.**

| **Models** | **Sensitivity (%)** | **Specificity (%)** | **Accuracy (%)** | **Positive Predictive Value (%)** | **Negative Predictive Value (%)** |
| --- | --- | --- | --- | --- | --- |
| **Model 1** | 79.63 | 70.83 | 75.49 | 75.44 | 75.56 |
| **Model 2** | 98.15 | 58.33 | 79.41 | 72.60 | 96.55 |
| **Model 3** | 90.74 | 75.00 | 83.33 | 80.33 | 87.81 |
| **Model 4** | 94.00 | 71.11 | 83.16 | 78.33 | 91.43 |

Model 1 was adjusted for age, sex, and body mass index (BMI), systolic blood pressure (SBP), diastolic blood pressure (DBP), diabetes duration and smoking status; Model 2 was adjusted for Model 1 and drugs, micro or macrovascular complications. Drugs include angiotensin-converting enzyme inhibitors (ACEI), angiotensin II receptor blockers (ARB), sodium-glucose cotransporter 2 inhibitors (SGLT-2i); glucagon-like peptide-1 receptor agonists (GLP-1 RAs), thiazolidinediones (TZD), insulin secretagogues, insulin, metformin or statins. Model 3 was adjusted for Model 2 and fasting blood glucose (FBG). Model 4 was adjusted for Model 3 and apolipoprotein A1 (ApoA1), apolipoprotein E (ApoE), lipoprotein(a) (Lp[a]).
